# Polar Wurtzite NbAlN: A Transition‐Metal Nitride Alloy for Polarization‐Engineered GaN Heterostructures

**DOI:** 10.1002/adma.74756

**Published:** 2026-09-03

**Authors:** Atsushi Kobayashi, Souta Kurogi, Tomoya Okuda, Riku Kikuchi, Yusuke Wakamoto, Kouei Kubota, Hikaru Sasaki, Kazuhisa Ikeda, Kanako Shojiki, Takahiro Kawamura, Toru Akiyama, Takehito Seki, Takuya Maeda

**Affiliations:** ^1^ Department of Materials Science and Technology Tokyo University of Science Tokyo Japan; ^2^ Department of Electrical Engineering and Information Systems The University of Tokyo Tokyo Japan; ^3^ Innovation Center for Semiconductor and Digital Future Mie University Mie Japan; ^4^ Graduate School of Engineering Mie University Mie Japan; ^5^ Institute of Engineering Innovation School of Engineering The University of Tokyo Tokyo Japan

**Keywords:** epitaxy, GaN, NbAlN, polar nitrides, two‐dimensional electron gas

## Abstract

The discovery of ferroelectric ScAlN sparked a search for new polar wurtzite alloys; however, incorporating transition metals remains a formidable challenge. Here, we demonstrate the synthesis of wurtzite‐phase NbAlN, a polar transition‐metal nitride alloy that is highly compatible with GaN‐based heterostructures. Despite the strong metallic character of elemental Nb, we achieved coherent epitaxial NbAlN films that preserve the wurtzite crystal structure and metal polarity of the underlying GaN, as directly visualized by annular bright‐field scanning transmission electron microscopy. Nb incorporation induces a monotonic lattice expansion and produces coherent Nb‐enriched nanoscale regions that are accommodated within the continuous wurtzite framework. Functionally, NbAlN barriers induce a substantial enhancement in the two‐dimensional electron gas density at the AlN/GaN heterointerface, consistent with polarization‐related carrier modulation by the metal‐polar NbAlN barrier. This study demonstrates that *d*‐electron transition metals can be integrated into polar nitride systems without compromising their functional asymmetry, making NbAlN a promising building block for polar nitride heterostructures and carrier‐density‐engineered GaN electronics.

## Graphical abstract

A novel polar wurtzite alloy, NbAlN, is demonstrated as a transition‐metal‐containing polar barrier for GaN heterostructures. Nb atoms form coherent nanoscale enrichment within the wurtzite lattice without disrupting metal polarity. This study demonstrates that transition metals can be integrated into polar nitrides to enable polarization‐related interfacial carrier modulation without intentional doping.

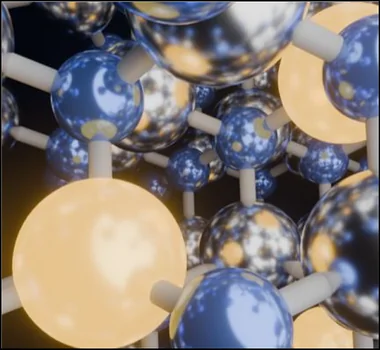

## 1 Introduction

Polar wurtzite III‐nitride semiconductors, including AlN and GaN, underpin modern high‐power and high‐frequency electronic devices owing to their wide bandgaps, high breakdown electric fields, and strong spontaneous and piezoelectric polarization [1, 2, 3]. In particular, polarization discontinuities at heterointerfaces, notably AlGaN/GaN heterojunctions, enable the formation of high‐density and high‐mobility two‐dimensional electron gases (2DEGs) without intentional doping, which form the operating basis of GaN‐based high‐electron‐mobility transistors (HEMTs) [4]. In addition to electronic transport, polarization in wurtzite nitrides governs diverse functionalities, including conventional piezoelectric actuation [5] and electro–optic modulation [6] to more advanced ferroelectric switching [7]. Consequently, expanding the palette of polar nitride materials that are highly compatible with GaN platforms remains a critical challenge.

A major breakthrough toward this goal was achieved using scandium aluminum nitride (ScAlN). Alloying AlN with Sc preserves the wurtzite structure even at high Sc concentrations while exhibiting pronounced lattice expansion, elastic softening, and a substantial enhancement of piezoelectric coefficients [8]. Recently, ScAlN thin films with moderate Sc composition exhibited robust room‐temperature ferroelectricity [9], establishing ScAlN as the first ferroelectric material that can be epitaxially grown directly on GaN [10]. Atomic‐resolution scanning transmission electron microscopy (STEM) further revealed that the large polarization in ScAlN originated from enhanced internal atomic displacements within the wurtzite lattice [11, 12]. These findings demonstrate that alloying AlN with selected metal elements can strongly modify its polarization‐related properties, thereby motivating the search for new polar nitride alloys beyond ScAlN [13, 14, 15, 16]. In this context, a broader class of emerging materials, commonly referred to as *X*AlN alloys (*X* = a metal element), has been reported. Recent studies have extended this family to include YAlN [17, 18, 19, 20, 21], BAlN [22, 23, 24, 25, 26], and even heterovalent alloys such as HfAlN [27], demonstrating that wurtzite ferroelectricity is a more general and widespread phenomenon.

Among potential alloying elements, Nb is an intriguing yet challenging candidate. Although competition between the tetrahedrally coordinated wurtzite AlN framework and alternative metal–N coordination environments can arise in various transition‐ and rare‐earth‐metal‐containing AlN alloys, Nb provides a particularly stringent case. Nb is a transition metal with partially filled *d* orbitals, multiple accessible valence states, and a strong tendency to form metallic rock‐salt NbN, a well‐known superconducting compound [28]. Its preference for octahedral coordination and metallic Nb─N bonding is therefore expected to compete strongly with the tetrahedrally coordinated AlN framework. At the same time, hexagonal Nb─N phases have been reported to exhibit structural compatibility with III‐nitride materials [29, 30], raising the fundamental question of whether Nb─N bonding environments can be incorporated into a polar wurtzite AlN lattice without disrupting its long‐range crystallographic order.

Previous studies on Nb‐containing AlN focused primarily on Mg─Nb co‐alloyed films designed to enhance the piezoelectric response. For example, Uehara et al. investigated Mg_0.39_Nb_0.25_Al_0.36_N and showed that a substantial piezoelectric enhancement was obtained through the simultaneous incorporation of Mg and Nb [31], with charge compensation involving Mg acceptors and multivalent Nb cations proposed to contribute to the resulting local lattice distortion [32]. These studies did not address whether codopant‐free ternary Nb*_x_* Al_1−_ *_x_* N could be stabilized as a single‐crystalline epitaxial wurtzite alloy, nor did they examine its crystallographic polarity or compatibility with GaN heterostructures. The incorporation of Nb into AlN while preserving the long‐range wurtzite order and polarity therefore remains an important fundamental question in nitride materials science. Throughout this work, NbAlN is used as a shorthand notation for the AlN‐based alloy Nb*_*x*_* Al_1−*x*_N, where *x* = Nb/(Nb + Al). Specific compositions are indicated by *x* or by the corresponding Nb cation percentage. Achieving single‐crystalline epitaxial NbAlN is essential because extended crystallographic coherence and an abrupt heterointerface are prerequisites for reliably relating its crystal polarity to polarization‐related carrier modulation in GaN‐based heterostructures. Establishing whether the polar stacking sequence is preserved across the NbAlN/GaN interface is particularly important because the direction of the polar axis determines the sign of the polarization‐bound charge and, consequently, the direction of interfacial carrier modulation.

Here, we report the first successful epitaxial growth of single‐crystalline wurtzite NbAlN thin films on GaN substrates and demonstrate that NbAlN represents a previously unrecognized class of metallic‐element–containing polar nitrides. Using reactive sputter epitaxy, we achieved high‐quality NbAlN films exhibiting smooth epitaxial surface morphologies after growth and excellent crystalline coherence with the underlying GaN. Systematic structural analyses revealed that the out‐of‐plane lattice parameter of NbAlN increased monotonically with increasing Nb composition while maintaining coherent in‐plane epitaxy on GaN. Atomic‐resolution STEM further revealed a unique microstructural feature: Nb‐rich nanoscale regions were accommodated within the AlN‐based framework, maintaining a continuous wurtzite lattice structure without forming grain boundaries.

Crucially, annular bright‐field (ABF) STEM directly visualized the polarity of NbAlN and demonstrated that the NbAlN layer inherited the metal‐polarity wurtzite structure of the GaN template. This observation provides atomic‐scale evidence that NbAlN preserves a well‐defined polar wurtzite stacking sequence, rather than forming a polarity‐disordered structure. Building on this structural foundation, we further demonstrate that epitaxial NbAlN, when employed as a barrier layer in AlGaN/GaN HEMT structures, leads to a substantial enhancement of the interfacial 2DEG density. The observed increase cannot be attributed solely to conventional carrier donation from Nb, but is more consistently explained by polarization‐related interfacial carrier modulation introduced by the metal‐polar NbAlN barrier.

Informed by previous studies on hexagonal Nb─N compounds, such as *β*‐Nb_2_N, and their high structural compatibility with AlN [29, 33, 34], this study extends the family of polar nitride alloys beyond ScAlN by demonstrating that Nb─N and Al─N coordination environments can be intermixed at the atomic scale within a single coherent wurtzite lattice structure. Therefore, NbAlN provides a promising transition‐metal‐containing polar barrier platform for GaN‐based nitride heterostructures, enabling polarization‐related carrier‐density modulation while maintaining continuity with established Nb‐based nitride epitaxy.

## 2 Results and Discussion

### 2.1 Epitaxial Growth of Wurtzite NbAlN on GaN

The epitaxial growth behavior of NbAlN films on GaN substrates was investigated using reflection high‐energy electron diffraction (RHEED) and atomic force microscopy (AFM), as shown in Figure 1. For Nb compositions of 11%, 16%, 23%, and 25%, the RHEED patterns recorded along the ⟨11$\bar{2}$0⟩ azimuth at the end of growth exhibited sharp, streaky features, indicating that the films retained smooth epitaxial surfaces and extended lateral coherence after deposition (Figure 1a). AFM images of representative films with Nb compositions of 16% and 25% further confirmed the smooth surface morphology. Well‐defined step‐and‐terrace structures, similar to those observed on the underlying GaN substrates, were consistently observed, along with low root‐mean‐square (RMS) surface roughness values (Figure 1b,c). These features indicate that NbAlN films retain smooth, 2D‐like epitaxial surface morphologies after growth for Nb compositions up to 25%. Because the present RHEED data were acquired at the end of growth, time‐resolved RHEED measurements would be required to determine whether step‐flow or layer‐by‐layer growth was maintained throughout the entire deposition process.

**FIGURE 1:**
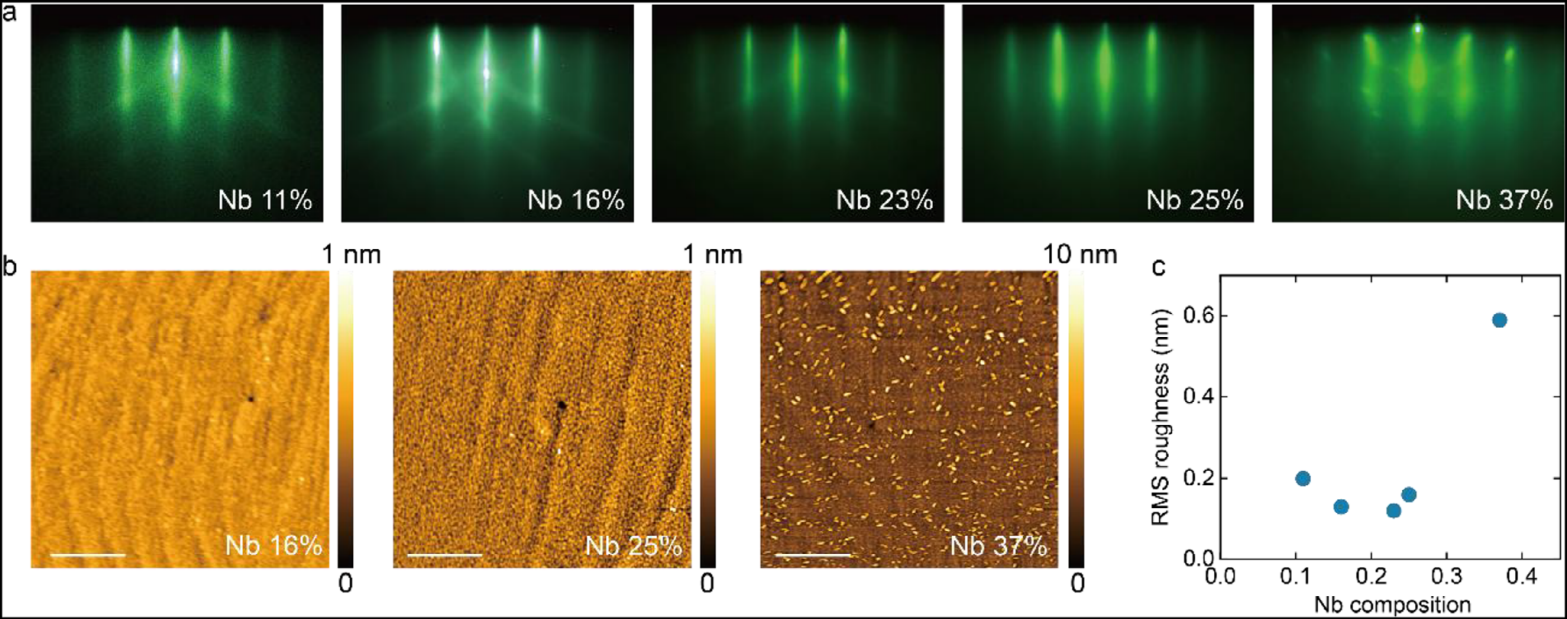
Epitaxial growth behavior of NbAlN films on GaN substrates. (a) RHEED patterns recorded along ⟨$11\bar{2}0$⟩ azimuth for Nb compositions of 11% (26 nm), 16% (23 nm), 23% (23 nm), 25% (24 nm), and 37% (27 nm). Well‐defined streaky patterns are observed for films with up to 25% Nb, whereas the 37% Nb film exhibits diffuse and spotty features. (b) AFM images of representative NbAlN films with Nb compositions of 16%, 25%, and 37%. Step‐and‐terrace morphologies are observed for Nb compositions up to 25%, whereas protruding features and a roughened surface are observed for 37% Nb. Scale bars correspond to 500 nm. (c) Root‐mean‐square surface roughness as a function of Nb composition, exhibiting a pronounced increase at high Nb composition.

By contrast, at a higher Nb composition of 37%, the RHEED pattern became diffuse and spotty, indicating a significant degradation in crystalline quality (Figure 1a). Correspondingly, AFM revealed a notably roughened surface with protruding features and a pronounced increase in RMS roughness (Figure 1b,c).

A wide‐range x‐ray diffraction (XRD) scan detected a weak non‐(000*l*) reflection consistent with wurtzite ($10\bar{1}2$) only for this composition, indicating the presence of a minor misoriented wurtzite component (Figure S1a). The coexistence of the protruding surface features and the misoriented wurtzite component is consistent with the formation of abnormally oriented grains. However, because XRD provides an area‐averaged structural response, a direct one‐to‐one correspondence between the individual AFM protrusions and the misoriented crystallographic component cannot be established.

These results indicate that maintaining high‐quality wurtzite epitaxial growth becomes increasingly difficult at 37% Nb under the present growth conditions.

### 2.2 Composition‐Dependent Lattice Evolution and Coherent Epitaxy of NbAlN

The lattice evolution and epitaxial strain state of the NbAlN films grown on GaN were analyzed by XRD. Figure 2a shows the 2*θ*/*ω* scans around the wurtzite 0002 diffraction peak for Nb compositions of 11%, 16%, 23%, 25%, and 37%. With increasing Nb composition, the 0002‐diffraction peak shifted systematically toward lower angles, indicating a continuous increase in the out‐of‐plane lattice parameter of the film with increasing Nb composition. Well‐defined interference fringes were observed for films with Nb compositions of up to 25%, indicative of high crystalline quality and smooth interfaces, whereas the diffraction intensity became weaker and broader at higher Nb composition.

**FIGURE 2:**
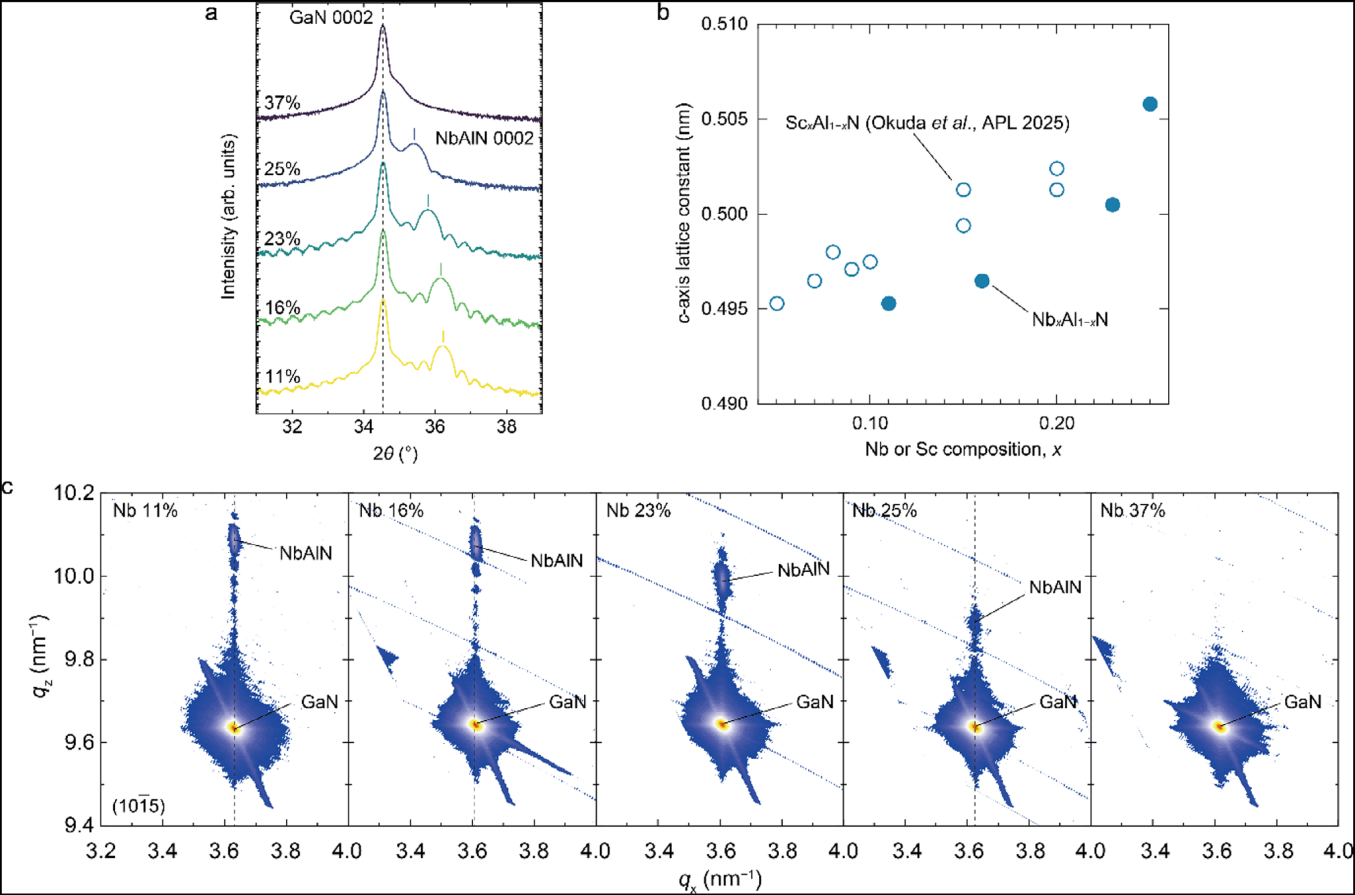
Composition‐dependent lattice evolution and coherent epitaxy of NbAlN films grown on GaN. (a) XRD 2*θ*/*ω* scans around wurtzite 0002 diffraction for Nb compositions of 11%, 16%, 23%, 25%, and 37%, exhibiting a systematic shift toward lower angles with increasing Nb composition. (b) Out‐of‐plane *c*‐axis lattice constant extracted from XRD as a function of Nb or Sc composition, *x*, for Nb*_x_* Al_1−_ *_x_* N in this work and Sc*_x_* Al_1−_ *_x_* N from our previous work [36]. The comparison represents the experimentally observed out‐of‐plane lattice response under GaN‐based epitaxial constraints rather than strain‐free bulk lattice parameters. (c) RSMs around the asymmetric ($10\bar{1}5$) reflection for representative Nb compositions of 11%, 16%, 23%, 25%, and 37%. Alignment of NbAlN and GaN peaks along *q*_x_ for Nb ≤ 25% indicates coherent epitaxy, whereas a shift toward lower *q*_z_ with increasing Nb composition indicates *c*‐axis lattice expansion.

The extracted *c*‐axis lattice constant is summarized as a function of the Nb composition as shown in Figure 2b. The *c*‐axis lattice parameter increased monotonically with increasing Nb composition, indicating a composition‐dependent lattice dilation in NbAlN. This systematic trend establishes NbAlN as an alloy system with tunable out‐of‐plane lattice parameters.

The epitaxial strain state was further examined via reciprocal space mapping (RSM) around the asymmetric (10$\bar{1}$5) reflection, as shown in Figure 2c. For Nb compositions between 11% and 25%, the NbAlN diffraction peaks were aligned with the GaN substrate peak along the in‐plane *q*_x_ direction, demonstrating coherent (pseudomorphic) epitaxial growth with a common in‐plane lattice spacing. Simultaneously, the NbAlN peak positions shifted toward lower *q*_z_ values with increasing Nb composition, which was consistent with the *c*‐axis lattice expansion deduced from the 2*θ*/*ω* scan results. At a higher Nb composition of 37%, the NbAlN peak intensity decreased significantly, indicating a degradation in the crystalline quality, although the peak position in the reciprocal space remained identifiable. Collectively, these XRD results revealed that NbAlN exhibited composition‐dependent lattice expansion while maintaining coherent in‐plane epitaxy on GaN up to an Nb composition of approximately 25%.

To further examine possible non‐wurtzite inclusions, wide‐range symmetric 2*θ*/*ω* scans from 25° to 80° were performed for NbAlN films with Nb compositions of 11%, 16%, 22%, 25%, and 37%, following the phase‐analysis strategy reported by Leone et al. (Figure S1a) [35]. No diffraction peaks attributable to a crystalline cubic Nb‐rich secondary phase were detected. For the 37% Nb film, a weak additional non‐000*l* reflection consistent with wurtzite ($10\bar{1}2$) was observed, indicating the presence of a minor misoriented wurtzite component.

In addition, *φ*‐scan measurements were performed for the 22%, 25%, and 37% Nb films at the nominal bulk *δ*‐NbN {002} diffraction condition (Figure S1b). No *φ*‐dependent peaks attributable to epitaxial *δ*‐NbN domains were detected at this specific condition. Because the lattice parameter of a hypothetical cubic Al─Nb─N phase may vary with composition, these *φ*‐scans exclude only cubic domains with lattice spacings close to that of bulk *δ*‐NbN. Combined with the wide‐range scans, the results show no detectable crystalline cubic secondary phase within the angular range and sensitivity of the present XRD analysis. However, trace cubic inclusions, peaks overlapping the dominant wurtzite or GaN reflections, or domains below the XRD detection limit cannot be completely excluded.

Although RSM analysis of NbAlN/GaN confirmed coherent epitaxy, it did not directly provide the strain‐free *a*‐axis lattice parameter of NbAlN, because the in‐plane lattice constant was constrained by the GaN substrate under pseudomorphic growth conditions. To obtain complementary information on the composition‐dependent in‐plane lattice evolution of NbAlN, an additional series of NbAlN films was epitaxially grown on AlN templates and analyzed by RSM (Figure S2). For Nb compositions up to 10%, the NbAlN layers remained fully coherent with AlN under the present thickness conditions, exhibiting an in‐plane lattice constant of approximately 0.311 nm, identical to that of AlN. For Nb compositions above 10%, the NbAlN layers showed partial strain relaxation, and the apparent *a*‐axis lattice parameter extracted from the RSMs increased with increasing Nb composition. In particular, the 13% and 17% NbAlN films had comparable thicknesses of 64 and 66 nm, respectively, and both showed partial relaxation, while the 17% film exhibited a larger apparent in‐plane lattice parameter. These results support the conclusion that the in‐plane lattice parameter of wurtzite NbAlN increases with Nb incorporation.

We note, however, that these values were obtained from partially relaxed films grown on AlN templates and should therefore not be interpreted as fully strain‐free bulk lattice parameters. Accordingly, Figure S2 is used here to show the qualitative trend of increasing in‐plane lattice parameter with increasing Nb composition, rather than to determine the exact strain‐free lattice constants of NbAlN or the exact lattice‐matching composition to GaN.

The lattice matching behavior of NbAlN on GaN was instead assessed from the thickness‐dependent cracking behavior and coherent‐growth‐window analysis of NbAlN films grown directly on GaN. As detailed in Supporting Note S3 and Figure S3, lower‐Nb‐composition films were more susceptible to tensile‐strain‐induced cracking, whereas the coherent crack‐free growth window expanded with increasing Nb composition. In particular, a 22% NbAlN film remained crack‐free at a thickness of 82 nm. This trend indicates that the tensile lattice mismatch between NbAlN and GaN decreases with increasing Nb composition and suggests that NbAlN approaches lattice matching to GaN at higher Nb compositions.

These results indicate that NbAlN is described as a wurtzite AlN‐based alloy, Nb*_x_* Al_1−_ *_x_* N, which could be discussed within the same conceptual framework as Sc*_x_* Al_1−_ *_x_* N. Within the epitaxial composition window, the lattice parameters varied monotonically with increasing Nb composition, demonstrating a systematic composition‐dependent modification of the wurtzite structure. This continuous lattice evolution was consistent with the alloy picture and fundamentally differed from the abrupt structural transition expected during macroscopic phase separation.

X‐ray photoelectron spectroscopy (XPS) indicated that the near‐surface Nb signal was dominated by Nb─N‐ or oxynitride‐related bonding, together with a higher‐binding‐energy component attributed to oxidized Nb species in the native surface layer; no clear metallic Nb° component was detected (Supporting Note S4 and Figure S4). Because the binding‐energy ranges associated with different Nb oxidation states overlap, the present XPS spectrum does not permit a unique quantitative separation of possible Nb^3+^ and Nb^5+^ contributions. Moreover, owing to the surface sensitivity of XPS, these measurements do not provide information on the spatial uniformity of Nb within the film or on the chemical state of the buried Nb‐rich nanoscale regions observed by STEM. Simultaneously, the multivalent nature of Nb could introduce local bonding rearrangements and charge‐compensation effects, leading to deviations from ideal Vegard behavior. Rather than invoking strict linear Vegard's law, we experimentally established a trend in which Nb incorporation expanded the wurtzite lattice in a controlled and continuous manner up to compositions that maintained high crystallinity and coherent epitaxial growth. In this respect, NbAlN expands the chemical space of polar AlN‐based wurtzite alloys toward transition‐metal incorporation while retaining epitaxial compatibility with GaN. Therefore, the XRD results indicate that NbAlN maintains a long‐range crystallographic order consistent with the wurtzite alloy structure, accommodating Nb incorporation through continuous lattice expansion.

Microscopic insights into the origin of the lattice expansion and phase stability were provided by first‐principles calculations performed for coherently strained NbAlN on GaN. As detailed in Supporting Note S5, the calculations confirmed that the wurtzite phase was energetically favored over the rock‐salt phase under GaN‐imposed coherent epitaxial strain across the experimentally relevant composition range. The calculated structures also reproduced the experimentally observed increase in the *c*‐axis lattice parameter with increasing Nb composition, demonstrating qualitative consistency between the theoretical and experimental results. Analysis of the relaxed atomic configurations under the same coherent constraint further showed that Nb─N bonds were longer than Al─N bonds and that Nb incorporation broadened the local bond‐length and bond‐angle distributions, particularly around Nb atoms (Supporting Note S6 and Figure S6). This differentiation of the local cation–N bonding environment is qualitatively similar to that reported for wurtzite ScAlN by Yazawa et al. [37], although a direct numerical comparison is not attempted because of the different computational conditions.

Notably, the first‐principles calculations supported an alloy‐based description of NbAlN, in which Nb atoms were incorporated as an integral part of the wurtzite lattice rather than acting as isolated dopants. The continuous evolution of the lattice parameters and local bonding environments underscores that NbAlN should be regarded as a polar AlN‐based wurtzite alloy, extending this materials family toward transition‐metal‐containing nitride compositions with partially filled *d* states. Furthermore, within the present coherent‐strain model, the calculated energy difference between the wurtzite and rock‐salt structures changes sign at a Nb composition near 60% on GaN, indicating an approximate energetic crossover (Figure S5). This value refers only to the relative phase energetics of the specific calculated configurations and should not be interpreted as an experimentally demonstrated growth or alloying range, or as evidence of favorable elastic, piezoelectric, ferroelectric, or electronic properties at such high Nb compositions. Indeed, the experimentally observed degradation of crystallinity and surface morphology at 37% Nb indicates that the practical growth window for high‐quality epitaxial wurtzite NbAlN under the present conditions is considerably narrower than the calculated structural range. Nevertheless, the calculations indicate that epitaxial stabilization can shift the relative energetic balance between the wurtzite and rock‐salt structures of NbAlN.

### 2.3 Atomic‐Scale Structure and Nb Distribution Revealed by STEM

The atomic‐scale structure of the epitaxial NbAlN films was investigated using cross‐sectional STEM. Figure 3a shows a low‐magnification low‐angle annular dark‐field (LAADF) STEM image of a representative Nb_0.23_Al_0.77_N film grown on a GaN/sapphire substrate. A continuous and well‐ordered wurtzite lattice was observed throughout the NbAlN layer, confirming its single‐crystalline nature. The NbAlN/GaN interface was abrupt and well defined, with no discernible interdiffusion or amorphous interlayers.

**FIGURE 3:**
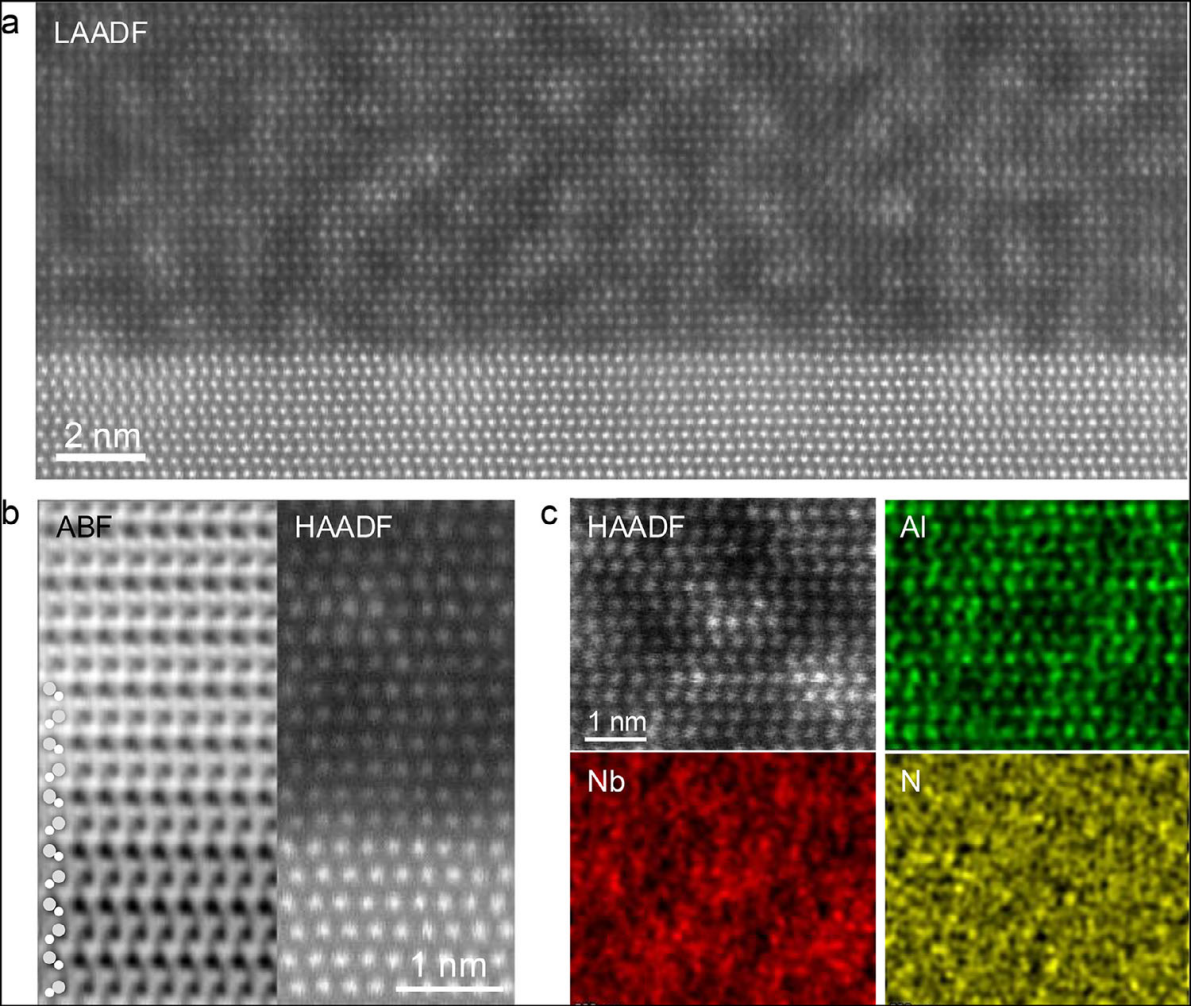
Atomic‐scale structural and compositional analyses of Nb_0.23_Al_0.77_N/GaN heterointerface. (a) Atomic‐resolution LAADF‐STEM image of Nb_0.23_Al_0.77_N film grown on GaN, confirming the wurtzite crystal structure and a coherent epitaxial relationship. (b) Magnified cross‐sectional STEM images of the NbAlN/GaN interface acquired using ABF and HAADF modes, revealing an atomically abrupt and coherent interface without observable secondary phases. Cation and N atoms are represented as gray and white circles, respectively. (c) HAADF‐STEM image and corresponding EDS elemental maps of Al, Nb, and N, demonstrating the nanoscale distribution of Nb within the wurtzite NbAlN lattice without macroscopic phase separation or secondary‐phase formation.

The ABF and high‐angle annular dark‐field (HAADF) STEM imaging were employed to determine the crystallographic polarity of the NbAlN layers. As shown in Figure 3b, the relative positions of the cation and anion atomic columns unambiguously indicate metal‐polar stacking near the NbAlN/GaN interface. The polarity direction was identical to that of the underlying GaN template, demonstrating that incorporation of Nb does not disrupt the polar stacking sequence of the wurtzite lattice structure. Additional atomic‐resolution ABF‐ and HAADF‐STEM observations near the upper surface region of the NbAlN layer revealed the same metal‐polar stacking sequence as that observed near the NbAlN/GaN interface, with no polarity inversion boundary observed in the analyzed near‐surface region (Figure S7). Together with the interfacial ABF‐STEM result, these observations indicate that the metal‐polar stacking sequence is maintained from the interface to the upper region of the film within the examined areas. To directly assess the possible presence and spatial distribution of oxygen at the buried interface, STEM energy‐dispersive x‐ray spectroscopy (EDS) elemental mapping and accumulated spectral analysis were performed across the NbAlN/GaN interface (Figure S8). A weak O‐*K* signal was observed in the accumulated spectra extracted from both the NbAlN layer and the NbAlN/GaN interfacial region. The corresponding O‐*K* map showed a broadly distributed weak signal across the TEM lamella, without pronounced oxygen enrichment either within the NbAlN layer or at the NbAlN/GaN interface. Because the origin of this weak O‐*K* signal cannot be determined uniquely from the present measurement, contributions from the TEM‐specimen surface or specimen preparation cannot be excluded. These results indicate the absence of spatially localized oxygen enrichment in the analyzed region, rather than the complete absence of oxygen. Together with the consistent metal‐polar stacking observed near the interface and near the upper surface region by ABF‐STEM, the lack of pronounced oxygen enrichment at the interface suggests that localized interfacial oxygen is unlikely to play a dominant role in determining the observed polarity under the present growth conditions. Previous studies have reported polarity inversion in sputtered nitride films induced by oxygen incorporation at interfaces and impurity‐related effects [38]. More recently, intentional oxygen incorporation was shown to change the as‐deposited polarity of sputtered Sc_0.27_Al_0.73_N films grown on [111]‐textured Pt bottom electrodes from N‐polar to metal‐polar through an intermediate mixed‐polarity regime [39]. This result demonstrates that oxygen can influence polarity selection during sputter growth when the underlying electrode does not provide a preexisting polar wurtzite stacking sequence. In the present study, however, the NbAlN films were epitaxially grown on Ga‐polar GaN, which provides a crystallographically defined metal‐polar template for polarity inheritance.

Accordingly, the present ABF‐STEM and STEM‐EDS results support the maintenance of metal polarity across the examined regions of the NbAlN layer under the present growth conditions. We also note that in our previous studies on sputtered ScAlN films grown on AlGaN/AlN/GaN heterostructures, no systematic polarity inversion behavior was observed [36, 40], suggesting that polarity inheritance can be maintained in these epitaxial sputtered nitride systems.

In addition to the long‐range crystallographic order, nanoscale compositional variations were observed within the NbAlN layer in the STEM images. Because the HAADF contrast scales approximately with the atomic number *Z*, these *Z*‐contrast variations were indicative of spatial inhomogeneity in the local chemical composition. EDS elemental mapping confirmed that the contrast‐enhanced regions correspond to Nb‐rich areas distributed throughout the NbAlN layer at the nanometer scale (Figure 3c).

These STEM observations verified the structural nature of NbAlN, revealing that it did not behave as a chemically homogeneous random alloy but instead exhibited nanoscale Nb enrichment while retaining extended lattice coherence.

### 2.4 Quantitative Analysis of Atomic Column Positions and Local Lattice Distortion

To quantitatively examine the relationship between local projected *Z*‐contrast and lattice distortion, we performed atomic‐column‐resolved analysis of the HAADF‐STEM image shown in Figure 4a using custom‐written analysis code. Initial atomic‐column candidates were identified by template matching, and their positions were refined to subpixel precision by 2D Gaussian fitting of the intensity distribution in the unfiltered HAADF‐STEM image. The fitted atomic‐column positions were then used to calculate the local *c*‐ and *a*‐axis lattice parameters according to the geometrical definitions shown in Figure 4b. The mean HAADF intensity within a local window centered on each fitted column was used as a qualitative measure of the projected *Z*‐contrast. Spatial maps of the resulting local *c*‐ and *a*‐axis lattice parameters are shown in Figure 4c,d, respectively.

**FIGURE 4:**
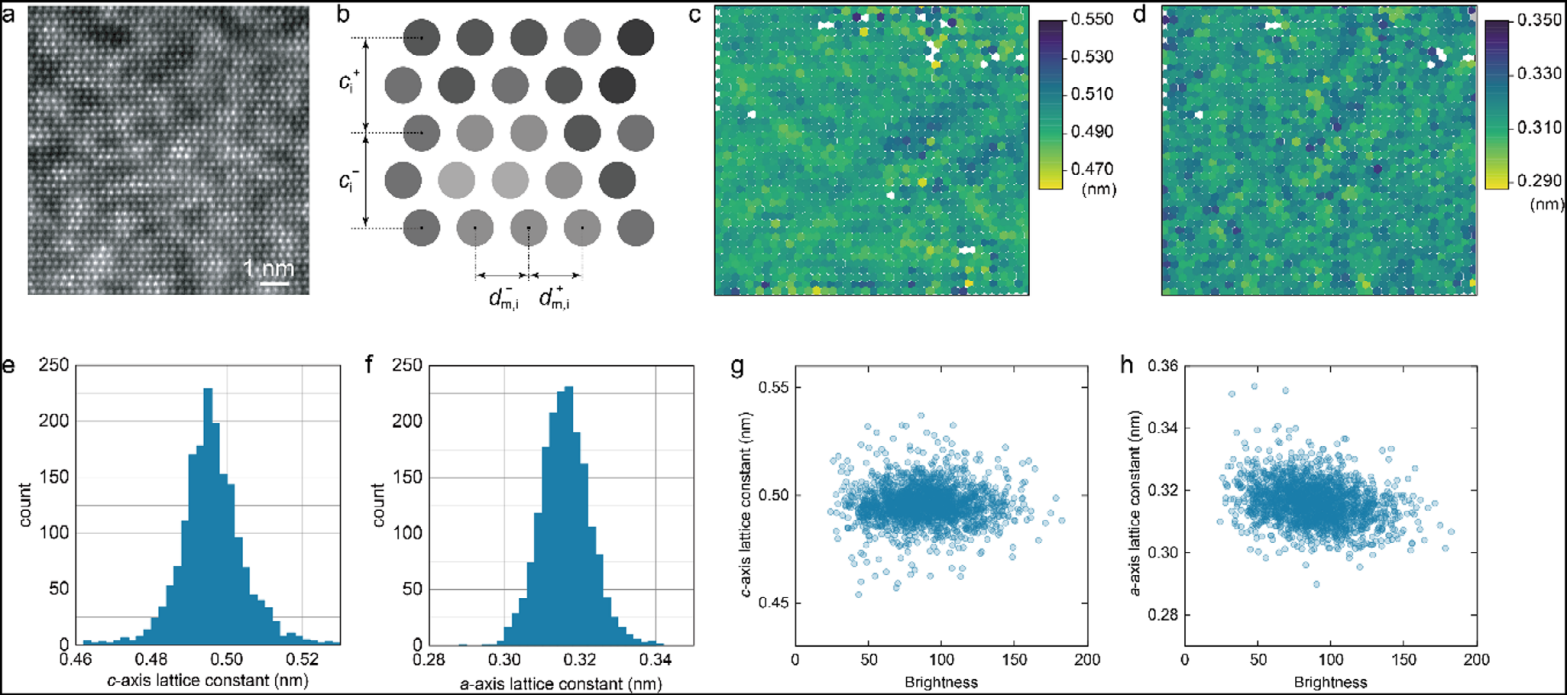
Quantitative analysis of atomic‐column positions and local lattice distortion in NbAlN. (a) Atomic‐resolution HAADF‐STEM image of Nb_0.23_Al_0.77_N used for atomic‐column analysis. (b) Schematic definition of the local lattice parameters from the fitted atomic‐column positions. When valid neighboring columns were available on both sides of a central column *i*, the local *c*‐axis lattice parameter was calculated as the average of the spacings to the crystallographically equivalent columns on the two opposite sides, $c_{i}=\left(c_{i}^{+}+c_{i}^{-}\right)\;/2$. For the [$11\bar{2}0]$ zone‐axis projection, the projected in‐plane spacing satisfies $d_{m}=\left(\sqrt{3}/2\right)\;a$; therefore, the local *a*‐axis lattice parameter was calculated as $a_{i}=\left(d_{m,i}^{+}+d_{m,i}^{-}\right)\;/\sqrt{3}$. Black dots indicate the fitted atomic‐column centers. (c, d) Spatial maps of the local *c*‐ and *a*‐axis lattice parameters, respectively, calculated from the fitted atomic‐column positions. (e, f) Histograms of the local *c*‐ and *a*‐axis lattice parameters, respectively. Both distributions exhibit a single dominant peak without a separately resolvable secondary component. (g, h) Relationships between the mean HAADF intensity of each atomic column and the local *c*‐ and *a*‐axis lattice parameters, respectively. The local *c*‐axis lattice parameter shows essentially no correlation with the mean HAADF intensity (*r* = 0.011), whereas the local *a*‐axis lattice parameter exhibits a weak negative correlation (*r* = −0.251).

To assess whether the Nb‐rich regions possess an independently relaxed lattice parameter, the local *c*‐ and *a*‐axis lattice parameters were statistically analyzed for the measurable atomic columns extracted from the HAADF‐STEM image in the analyzed region. The histogram of the local *c*‐axis lattice parameter exhibited a single, well‐defined peak without noticeable asymmetry or secondary features, indicating that no separately resolvable *c*‐axis lattice‐parameter population is detected within the spatial resolution and statistical limits of the present analysis (Figure 4e). Similarly, the local *a*‐axis lattice parameter exhibited a single dominant distribution without a separately resolvable secondary component (Figure 4f).

Furthermore, no systematic correlation was observed between the atomic‐column intensity, which served as a qualitative projected *Z*‐contrast‐based proxy for local Nb enrichment, and the local *c*‐axis lattice parameter (Figure 4g). In contrast, a weak negative correlation was observed between the atomic‐column intensity and the local *a*‐axis lattice parameter (Pearson *r* = −0.251), indicating a slight tendency toward reduced in‐plane spacing in the HAADF‐bright regions (Figure 4h). The local *c*‐axis lattice parameter showed essentially no correlation with the atomic‐column intensity (Pearson *r* = 0.011). These results suggest that the Nb‐enriched regions may be accompanied by weak anisotropic local lattice distortion while remaining crystallographically coupled to the surrounding wurtzite framework.

The HAADF‐bright regions were spatially consistent with the Nb‐rich regions identified by STEM‐EDS, supporting the use of HAADF intensity as a qualitative indicator of local Nb enrichment. It should be noted, however, that HAADF intensity is a projected signal and can be influenced by specimen thickness, channeling, and local specimen conditions. Therefore, the absence of a clear correlation between HAADF intensity and local *c*‐axis parameter does not indicate the absence of Nb enrichment. Rather, the combined *c*‐ and *a*‐axis analyses indicate that the Nb‐rich nanoscale regions do not possess a separately resolvable lattice‐parameter population corresponding to an independently relaxed phase.

These results are physically consistent with the composition‐dependent lattice expansion observed by XRD. The XRD results show that the average *c*‐axis lattice parameter of NbAlN increases with increasing Nb composition, whereas the STEM analysis probes spatial variations within a single coherently grown film. Thus, Nb incorporation expands the average wurtzite lattice at the film level, but the Nb‐rich nanoscale regions within the film remain crystallographically coupled to the surrounding matrix and share a common projected *c*‐axis periodicity. At the same time, the weak negative correlation observed for the local *a*‐axis lattice parameter suggests that limited in‐plane structural accommodation may occur in the HAADF‐bright regions. This combination of a common projected *c*‐axis periodicity and weak in‐plane variation is consistent with the clear XRD interference fringes, coherent RSM features, and the absence of observable grain boundaries or secondary phases in the analyzed regions of atomic‐resolution STEM.

Atomic‐resolution STEM therefore revealed Nb‐rich nanoscale regions embedded within a continuous AlN‐based wurtzite framework. While such compositional modulation may visually resemble decomposition‐like contrast often observed in metastable transition‐metal nitride alloys, the present analysis indicates an important difference. In conventional decomposition, compositionally distinct regions are often accompanied by lattice relaxation, secondary‐phase formation, or a split or broadened distribution of local lattice spacings. In contrast, the present Nb‐rich regions retain a common projected *c*‐axis periodicity with the surrounding matrix, show only a weak tendency toward local in‐plane contraction, and exhibit no separately resolvable lattice‐parameter population in either crystallographic direction. These observations do not support the presence of an independently relaxed Nb‐rich secondary phase within the spatial resolution and statistical limits of the present analysis.

The origin of the pronounced nanoscale compositional modulation is not yet fully understood. One possible interpretation is that the distinct bonding and coordination preferences of Nb─N and Al─N create an energetic tendency toward local compositional modulation. The formation of Nb‐rich and Al‐rich regions may permit partial accommodation of the different local bonding environments and may reduce local chemical and elastic energies while preserving the overall wurtzite framework. However, the present results do not establish whether local stress accommodation is the dominant driving force or whether the modulation arises from a specific decomposition mechanism.

The nanoscale dimensions and crystallographic coherence of these regions are likely facilitated by the highly nonequilibrium nature of sputter epitaxy and by the epitaxial constraint imposed by the GaN substrate. These factors can limit long‐range atomic redistribution and inhibit macroscopic phase separation and transformation into equilibrium secondary phases, thereby kinetically arresting the compositional modulation within a continuous wurtzite lattice.

Because atomic‐resolution STEM and STEM‐EDS analyses were performed only for the Nb_0.23_Al_0.77_N film, the composition dependence of the nanoscale compositional modulation cannot be determined from the present data. We tentatively expect that the modulation is relatively weak at low Nb compositions and becomes more pronounced with increasing Nb incorporation, owing to the increasing chemical and structural mismatch between the preferred Nb─N and Al─N bonding environments. Below a composition‐dependent stability limit, such compositional modulation may remain coherently accommodated within the wurtzite lattice under the epitaxial constraint imposed by GaN. The Nb_0.23_Al_0.77_N film examined by STEM is considered to lie within this coherent wurtzite regime, whereas the substantial degradation in crystalline quality observed at an Nb composition of 37% suggests that this composition lies beyond the stability range for high‐quality wurtzite NbAlN growth under the present conditions. Systematic composition‐dependent STEM, STEM‐EDS, and local lattice‐parameter analyses will be required to determine how the amplitude and characteristic length scale of the compositional modulation evolve with Nb composition and whether secondary phases emerge at higher Nb compositions.

Previous studies have demonstrated that Nb─N compounds can adopt hexagonal structures, such as *β*‐Nb_2_N [29, 33, 34, 41, 42, 43], which are structurally compatible with wurtzite AlN. From this perspective, the observed Nb‐rich nanoordering in NbAlN may reflect local Nb─N coordination motifs that could be accommodated within the AlN‐based wurtzite framework without disrupting its long‐range crystallographic order or polarity. Rather than representing a conventional precipitated secondary phase, these Nb‐enriched regions may serve as a coherent structural‐accommodation motif that helps incorporate Nb─N local coordination into the AlN‐based wurtzite framework over a finite compositional range.

The key outcome of this study is the direct determination of the polarity of the epitaxial Nb_0.23_Al_0.77_N film examined here using ABF STEM. Atomic column–resolved images provided unambiguous evidence that Nb_0.23_Al_0.77_N maintained metal‐polar stacking with a polarity direction identical to that of the underlying GaN template. This observation revealed that incorporation of Nb at a composition of 23% and for the film thickness examined here did not disrupt the polar stacking sequence of the wurtzite lattice, even though Nb is a transition metal with partially filled *d* orbitals and multiple accessible valence states.

Preservation of polarity has critical implications for understanding the structural properties of NbAlN. In wurtzite nitrides, polarity arises from the long‐range stacking order of tetrahedrally coordinated cation–anion bilayers. The present results indicate that the long‐range polar stacking is preserved by the epitaxially continuous AlN‐based wurtzite framework, despite the local incorporation of Nb. Accordingly, the Nb_0.23_Al_0.77_N film examined in this study forms a *c*‐axis‐oriented wurtzite alloy with a well‐defined metal‐polar stacking direction, despite the incorporation of a transition metal.

Notably, the Nb_0.23_Al_0.77_N film retained the metal‐polar stacking sequence of the underlying Ga‐polar GaN template. This observation shows that, under the present sputter‐epitaxy conditions and for the composition and thickness investigated, Nb incorporation did not induce a detectable polarity inversion across the analyzed region. Although this behavior is consistent with polarity inheritance commonly observed in epitaxial III‐nitride heterostructures [4, 44, 45], the present result does not establish that the polarity of NbAlN is determined solely by the substrate polarity. Previous studies of sputtered ScAlN/GaN have shown that polarity may also depend on growth conditions and film thickness, including the formation of polarity‐inverted or mixed‐polarity regions. Systematic growth on Ga‐polar and N‐polar templates over a wider thickness range will therefore be required to determine the respective roles of substrate polarity and sputter‐growth conditions in NbAlN. The direct confirmation of polarity preservation in the Nb_0.23_Al_0.77_N film supports the potential of NbAlN as a building block for polar heterostructures on GaN platforms and for the integration of transition‐metal‐containing nitrides into polarity‐engineered device architectures.

The polarity was directly determined only for the approximately 25‐nm‐thick Nb_0.23_Al_0.77_N film analyzed by ABF STEM. For films with Nb compositions up to 25%, the epitaxial wurtzite RHEED pattern suggests that no major crystallographic reorientation occurred; however, RHEED does not provide a direct determination of polarity. The polarity of films with other Nb compositions and of thicker films therefore remains to be established experimentally. In particular, the substantial degradation in the RHEED pattern and surface morphology of the 37% Nb film prevents a reliable assignment of its polarity from the present measurements.

### 2.5 Enhancement of 2DEG by NbAlN Barrier Layers

The effect of NbAlN barrier layers on the sheet carrier density of the 2DEG was investigated at room temperature using Hall effect measurements in van der Pauw geometry. The heterostructure studied consisted of NbAlN/Al_0.2_Ga_0.8_N/AlN/GaN, where the NbAlN layer served as a polar barrier layer in combination with AlGaN and AlN. The Al_0.2_Ga_0.8_N/AlN/GaN heterostructure template used in this study was grown by metal–organic chemical vapor deposition (MOCVD) on semi‐insulating 6H‐SiC substrates (see Experimental Section for details). The relatively low sheet carrier density in the reference AlGaN/AlN/GaN heterostructure can be attributed to the thin (∼3 nm) and low‐Al‐content (20%) AlGaN barrier layer, despite the presence of the ultrathin (∼1 nm) AlN interlayer. This barrier design reduces the overall polarization discontinuity and hence the 2DEG density. This structure was intentionally designed to provide a clear baseline for evaluating the enhancement effect induced by NbAlN barrier layers. RSM confirmed that the AlGaN/AlN/GaN layers remained coherently strained to the GaN layer and that the NbAlN barrier layer grew coherently on the underlying heterostructure. Consistent with this high structural coherence, AFM images revealed a smooth surface morphology exhibiting clear atomic step‐and‐terrace structures (Figure 5a). This observation confirms that structurally high‐quality wurtzite NbAlN layers can be realized on AlGaN templates with minimal surface roughening.

**FIGURE 5:**
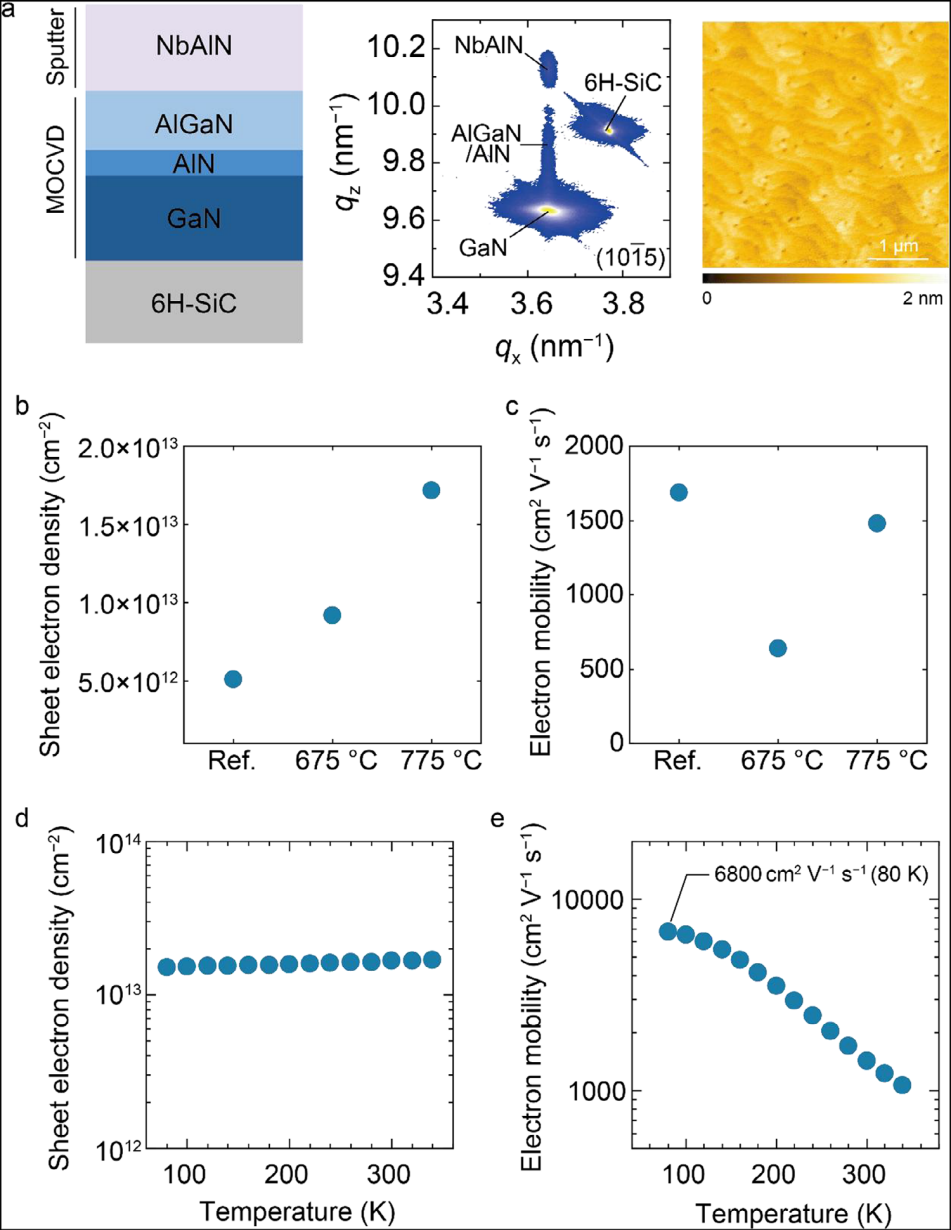
Enhancement of 2DEG formation by NbAlN barrier layers. (a) Schematic illustration, RSM, and AFM image of the NbAlN/AlGaN/AlN/GaN/6H‐SiC heterostructure used for electrical characterization. (b) Sheet carrier density at room temperature for reference structure without NbAlN and for heterostructures incorporating NbAlN barriers grown at 675°C and 775°C. (c) Room‐temperature electron mobility for the same set of heterostructures. (d) Temperature dependence of sheet carrier density for the NbAlN barrier with 10% Nb, showing nearly constant carrier density upon cooling. (e) Temperature dependence of electron mobility for the same sample. The mobility reaches 6800 cm^2^ V^−1^ s^−1^ at 80 K, indicating the formation of a high‐quality 2DEG channel.

For the control samples without NbAlN, the sheet electron density was approximately 5.1 × 10^12^ cm^−2^. By contrast, the introduction of a NbAlN barrier with a Nb composition of 10% and a thickness of 13 nm led to a significant increase in the 2DEG density. For NbAlN grown at 775°C, the sheet carrier density increased to 1.7 × 10^13^ cm^−2^, representing more than a threefold increase relative to the reference structure. When NbAlN was grown at 675°C, the 2DEG density was enhanced, reaching approximately 9 × 10^12^ cm^−2^ (Figure 5b). These results demonstrate that the presence of a NbAlN barrier significantly enhances electron accumulation at the heterointerface, with the magnitude of the enhancement dependent on the growth temperature of the barrier layer. Similar carrier‐density enhancements associated with polarization discontinuities have been extensively reported for ScAlN/GaN HEMTs, where the increased interface charge has enabled high‐current operation [46, 47, 48].

The electron mobility exhibited different behaviors. Although the reference structure exhibited a room‐temperature mobility of approximately 1690 cm^2^ V^−1^ s^−1^, the mobility decreased to 644 cm^2^ V^−1^ s^−1^ for NbAlN grown at 675°C. By contrast, NbAlN grown at 775°C maintained a relatively high mobility of 1485 cm^2^ V^−1^ s^−1^ (Figure 5c). These results indicate that the NbAlN barriers enable a substantial enhancement in the 2DEG density, whereas the resulting transport properties are sensitive to the growth conditions of the NbAlN layer. To further assess the origin and quality of the electron channel, temperature‐dependent Hall effect measurements were performed for the NbAlN barrier with 10% Nb. As shown in Figure 5d, the sheet electron density remains nearly constant upon cooling, indicating that the carriers are not thermally activated. This behavior suggests that the observed electron density is not dominated by unintentional donor impurities such as Si or O, but is more consistent with polarization‐related carrier accumulation. Notably, the electron mobility increases upon cooling and reaches 6800 cm^2^ V^−1^ s^−1^ at 80 K (Figure 5e), highlighting the high quality of the 2DEG channel in the NbAlN‐based heterostructure. Capacitance–voltage (*C*–*V*) measurements were not performed because significant leakage currents prevented reliable capacitance extraction in the present heterostructures.

The enhanced 2DEG density observed upon incorporating the NbAlN barrier cannot be attributed solely to strain‐induced changes in the underlying heterostructure. RSM confirmed that the AlGaN/AlN/GaN layers remained coherently strained to GaN and that the NbAlN layer grew coherently on the underlying structure. Consequently, the strain state of the heterostructure was largely preserved after incorporating NbAlN.

The observed carrier‐density enhancement is consistent with a modification of the effective boundary condition associated with the metal‐polar wurtzite NbAlN barrier. In III‐nitride heterostructures, the 2DEG density is governed by the electrostatic boundary condition associated with polarization discontinuities at polar heterointerfaces. Therefore, replacing the conventional barrier with a metal‐polar NbAlN layer can modify the effective interfacial charge distribution and thereby increase the electron density at the AlN/GaN interface.

A simple electrostatic estimate provides a quantitative measure of this carrier modulation. Using *Δσ*_eff_ = *qΔn*_s_, the increase in sheet electron density corresponds to an additional effective sheet charge density. Compared with the reference AlGaN/AlN/GaN heterostructure, the NbAlN barrier grown at 775°C increased the sheet electron density from approximately 5.1 × 10^12^ cm^−2^ to 1.7 × 10^13^ cm^−2^, corresponding to an additional effective sheet charge density of approximately 1.9 µC cm^−2^. For the NbAlN barrier grown at 675°C, the corresponding increase was approximately 0.63 µC cm^−2^. These values should be regarded as effective charge modulation estimated from Hall effect measurements, rather than direct measurements of the intrinsic polarization constants of NbAlN.

This interpretation is supported by the experimentally confirmed metal polarity of NbAlN and by the absence of intentional doping in the NbAlN barrier layer. In addition, the nearly temperature‐independent sheet carrier density observed in the low‐temperature Hall effect measurements suggests that the enhanced electron density is not dominated by thermally activated donor impurities. Nevertheless, because direct measurements of polarization constants and quantitative impurity profiling were not performed in this study, contributions from residual impurities cannot be completely excluded. Further studies involving *C*–*V* profiling, time‐of‐flight secondary ion mass spectrometry, and detailed electrostatic modeling would be valuable for quantitatively separating polarization‐related and impurity‐related contributions.

We note that NbAlN layers grown directly on GaN without the AlGaN/AlN interlayers did not exhibit measurable electron conduction attributable to a 2DEG, as confirmed by Hall effect measurements. The origin of this behavior cannot be assigned uniquely to residual impurities at the regrowth interface. Several additional factors may influence carrier accumulation in the direct NbAlN/GaN structure. Low‐Nb‐composition NbAlN films are coherently tensile strained on GaN, which is expected to modify the piezoelectric contribution to the total polarization. However, because the spontaneous polarization, piezoelectric coefficients, and band offsets of NbAlN have not yet been determined, the resulting polarization discontinuity and carrier‐confinement potential cannot presently be quantified. Moreover, if the nanoscale compositional modulation observed within the NbAlN layer extends to the initial interface region, it may produce lateral variations in polarization, band alignment, and interface‐state density. Energetic species generated during sputter deposition may also introduce point defects or interfacial mixing in the near‐surface region of the GaN template, potentially compensating polarization‐induced charge or trapping carriers.

The directly grown NbAlN/GaN structure also differs fundamentally from the NbAlN/AlGaN/AlN/GaN heterostructure used for the transport measurements in Figure 5, in which the electron channel is formed at the AlN/GaN interface. The presence of the AlN and AlGaN interlayers changes the polarization discontinuity, band alignment, interface abruptness, and electrostatic boundary conditions. Residual Si or O at the sputter‐regrowth interface remains another possible contribution, although its role cannot be isolated from the present transport measurements. Therefore, the absence of a measurable 2DEG in directly grown NbAlN/GaN is likely governed by a combination of strain‐related, interfacial, compositional, defect‐related, and electrostatic factors rather than by a single impurity mechanism. The transport properties of heterostructures incorporating more closely lattice‐matched NbAlN layers, expected at Nb compositions of approximately 25% or higher, have not yet been investigated. Systematic transport measurements across this composition range will be required to determine the influence of epitaxial strain on carrier accumulation and mobility.

The observed dependence of electron mobility on the growth temperature revealed a decrease from 1690 cm^2^ V^−1^ s^−1^ in the reference to 644 cm^2^ V^−1^ s^−1^ for NbAlN grown at 675°C but recovering to 1485 cm^2^ V^−1^ s^−1^ at 775°C. This aligns with our previous findings in ScAlN/AlGaN heterostructures [48]. In the ScAlN system, higher growth temperatures improved mobility, likely by suppressing oxidation or defect formation at the regrowth interface between the air‐exposed AlGaN template and the sputtered barrier layer. A similar mechanism may also contribute to the growth‐temperature‐dependent mobility observed in the NbAlN system, where the quality of the regrowth interface may be a key factor governing the transport properties. Future implementation of an all‐sputtered, vacuum‐integrated growth process for the entire heterostructure would eliminate air exposure at the interface, enabling a more rigorous assessment of the intrinsic transport limits of NbAlN‐based heterostructures.

Consequently, these results establish NbAlN as a promising transition‐metal‐containing polar barrier for GaN‐based heterostructures.

Although the present heterostructures are relevant to GaN HEMT barrier design, full transistor devices were not fabricated in this study. Therefore, transfer characteristics, output characteristics, gate leakage, and contact resistance were not evaluated. Instead, this work focuses on material‐ and heterostructure‐level transport properties, including sheet carrier density, electron mobility, sheet resistance, and temperature‐dependent Hall response. These results provide the first assessment of NbAlN as a transition‐metal‐containing polar barrier material for high‐conductivity GaN electronic heterostructures. Future work will involve full transistor fabrication with optimized ohmic contacts and gate stacks to evaluate the device‐level performance of NbAlN‐based HEMTs. In particular, pulsed *I*–*V* and large‐signal *I*–*V* measurements will be important for assessing possible trapping behavior and for clarifying the influence of Nb‐rich nanoscale regions on practical device operation.

### 2.6 Stabilization Mechanism and Comparison With ScAlN

Based on the structural and electrical findings presented above, the fundamental nature of NbAlN alloy systems was assessed in comparison with the established ScAlN system. Both NbAlN and ScAlN can be viewed as AlN‐based polar wurtzite alloys in which the wurtzite structure is retained over a finite composition window, the lattice parameters evolve systematically with alloy composition, and the metal polarity can be preserved when grown epitaxially on GaN. These common features support the classification of NbAlN within the broader family of polar AlN‐based wurtzite alloys, rather than as a distinct or anomalous material class.

To provide a quantitative structural benchmark against ScAlN, the composition‐dependent *c*‐axis lattice constants of NbAlN were compared with those of ScAlN from our previous work (Figure 2b). This comparison represents the experimentally observed out‐of‐plane lattice response under GaN‐based epitaxial constraints, rather than strain‐free bulk lattice parameters. Both ScAlN and NbAlN exhibit systematic *c*‐axis expansion with increasing alloy composition, confirming that NbAlN follows a composition‐dependent lattice evolution characteristic of AlN‐based wurtzite alloys.

A notable difference is observed in the absolute *c*‐axis lattice constant at comparable alloy compositions. At similar *X* (Sc or Nb) composition, the *c*‐axis lattice constant of NbAlN is smaller than that of ScAlN. This difference can be understood by considering the different lattice‐matching compositions of the two alloys with GaN. In ScAlN, the lattice‐matched composition to GaN has been reported to be approximately 9%–11% Sc [49]. Therefore, ScAlN films with higher Sc composition are expected to experience in‐plane compressive strain when coherently constrained by GaN, leading to *c*‐axis expansion through elastic coupling. In contrast, the growth‐window analysis of NbAlN on GaN (Supporting Note S3 and Figure S3) indicates that the critical thickness for crack‐free coherent growth increases with increasing Nb composition and that a 22% NbAlN film remains crack‐free even at a thickness of 82 nm. This trend indicates that the lattice mismatch between NbAlN and GaN is reduced with increasing Nb composition and suggests that NbAlN approaches lattice matching to GaN at compositions higher than 22% Nb. The higher lattice‐matching composition of NbAlN compared with ScAlN indicates that Nb incorporation produces a smaller average in‐plane lattice expansion per alloy fraction. This difference should not be interpreted from atomic number alone, because the lattice response depends on the effective ionic size, local coordination, and bonding character of the alloying cation. The smaller effective ionic size of Nb relative to Sc for comparable coordination states may contribute to the weaker in‐plane expansion. In addition, the coherent Nb‐rich nanoscale compositional modulation observed by STEM may allow local Nb─N coordination and structural mismatch to be accommodated without a proportional increase in the average in‐plane lattice parameter.

Accordingly, NbAlN remains on the tensile‐strain side up to a higher *X* composition than ScAlN when coherently grown on GaN. This tensile in‐plane strain tends to contract the out‐of‐plane *c*‐axis through elastic coupling, providing a physically consistent explanation for the smaller *c*‐axis lattice constant of NbAlN compared with ScAlN at similar alloy compositions. This comparison clarifies that NbAlN occupies a distinct epitaxial strain regime from ScAlN and provides access to a higher‐*X*‐composition coherent growth window on GaN.

Despite this structural similarity, the microscopic accommodation mechanisms in NbAlN and ScAlN are distinct. ScAlN has generally been discussed as a relatively homogeneous AlN‐based alloy in which Sc incorporation modifies the wurtzite lattice through elastic softening and local coordination changes. In contrast, NbAlN exhibits a self‐organized nanostructure, as revealed by STEM observations, where coherent Nb‐enriched nanoscale regions are embedded within a continuous AlN‐based wurtzite framework. Quantitative atomic column analysis (Figure 4) showed that these Nb‐enriched regions share a common projected *c*‐axis periodicity with the surrounding matrix and do not form an independently relaxed phase with a distinct *c*‐axis lattice parameter. Therefore, the nanoscale Nb enrichment is interpreted not as conventional relaxed phase separation, but rather as coherent nanoordering within the wurtzite NbAlN lattice.

This coherent nanoordering provides a plausible structural‐accommodation motif for incorporating Nb─N local coordination into the AlN‐based wurtzite framework. Importantly, this interpretation does not imply the absence of local strain or local bonding relaxation. Rather, it indicates that such relaxation is accommodated within a crystallographically continuous wurtzite lattice without disrupting long‐range order or metal polarity. From this perspective, NbAlN and ScAlN are complementary polar AlN‐based alloys with distinct stabilization mechanisms: ScAlN expands the polar nitride materials space through Sc‐induced lattice softening and ferroelectricity, whereas NbAlN expands it toward transition‐metal‐containing wurtzite alloys through coherent nanoscale ordering.

The optical bandgap and electronic functionality of NbAlN were further benchmarked against ScAlN‐based materials and GaN heterostructures (Figure 6). Figure 6a compares the optical bandgaps of NbAlN films epitaxially grown on bulk GaN with those of ScAlN films reported in our previous work [50]. The NbAlN films exhibited optical bandgaps of 5.78 and 5.68 eV for Nb compositions of 13% and 21%, respectively. These values demonstrate that NbAlN retains a wide‐bandgap character exceeding 5.6 eV despite the incorporation of a *d*‐electron transition metal. For comparison, ScAlN exhibited bandgaps of 5.66, 5.59, and 5.46 eV for Sc compositions of 7%, 10%, and 14%, respectively. In the measured composition ranges, the NbAlN films retain bandgaps comparable to or larger than those reported for ScAlN films. This result is important because it indicates that Nb incorporation strongly modifies the lattice and local bonding environment while preserving a wide‐bandgap character suitable for GaN barrier applications. We note, however, that detailed band offsets and band alignment with GaN were not determined in the present study and remain important subjects for future work.

**FIGURE 6:**
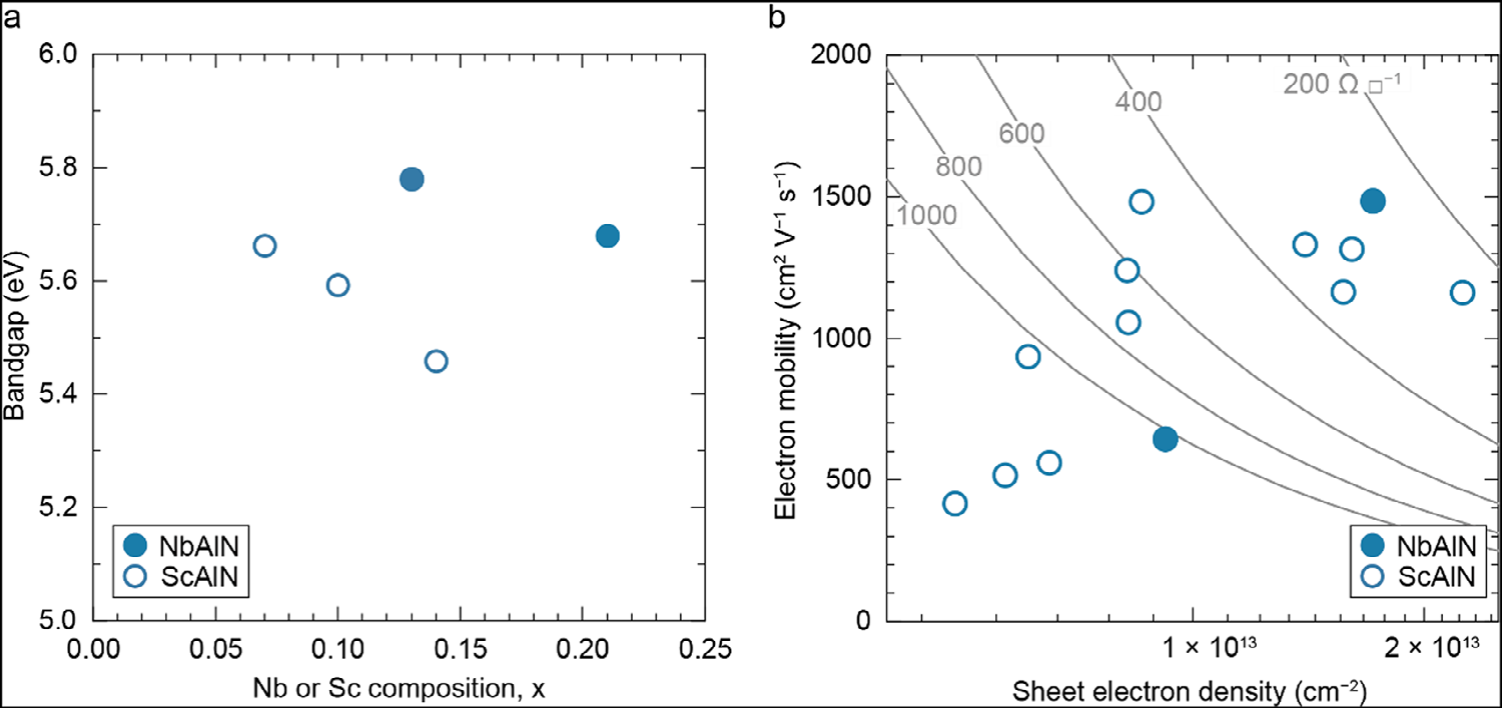
Quantitative benchmark of NbAlN against ScAlN. (a) Optical bandgap as a function of Nb or Sc composition. The NbAlN data were obtained from spectroscopic ellipsometry of epitaxial NbAlN films grown on bulk GaN, and the ScAlN data are from our previous work. NbAlN retains a wide optical bandgap of 5.68–5.78 eV for Nb compositions of 13%–21%. (b) Benchmark of room‐temperature Hall transport properties for NbAlN/AlGaN/AlN/GaN and ScAlN/AlGaN/AlN/GaN heterostructures. Electron mobility is plotted as a function of sheet electron density. The NbAlN data are from the present work, and the ScAlN data are from reported ScAlN heterostructures [36, 40, 51]. The contour lines indicate constant sheet resistance, calculated using *R*_sh_ = (*qn*_s_ *µ*)^−1^. The optimized NbAlN barrier grown at 775°C achieves a high sheet electron density and high mobility, placing it in a low‐sheet‐resistance regime comparable to high‐performing reported ScAlN‐based heterostructures.

The room‐temperature Hall transport properties were also benchmarked against ScAlN‐based GaN heterostructures [36, 40, 51] (Figure 6b). The NbAlN barriers in the present work showed sheet electron densities of 0.92 and 1.7 × 10^13^ cm^−2^ with electron mobilities of 644 and 1485 cm^2^ V^−1^ s^−1^ for the barriers grown at 675°C and 775°C, respectively. These values correspond to sheet resistances of approximately 1050 and 245 *Ω*▫^−1^, respectively. In comparison, reported ScAlN/AlGaN/AlN/GaN heterostructures cover a broad range of transport properties, with sheet electron densities of 0.49–2.2 × 10^13^ cm^−2^ and mobilities of 416–1484 cm^2^ V^−1^ s^−1^. Although the highest reported ScAlN/AlGaN/AlN/GaN carrier density exceeds that obtained in the present NbAlN heterostructures, the optimized NbAlN barrier grown at 775°C simultaneously achieves high sheet electron density and high mobility, placing it in a low‐sheet‐resistance regime comparable to high‐performing reported ScAlN‐based heterostructures. In contrast, the NbAlN barrier grown at 675°C exhibits a higher sheet resistance because of its lower carrier density and mobility, highlighting the importance of growth‐temperature optimization and interfacial quality for realizing the full transport potential of NbAlN barriers.

These benchmarks indicate that NbAlN is not merely a structurally interesting transition‐metal‐containing wurtzite alloy, but also a wide‐bandgap and practically competitive polar barrier material for high‐conductivity GaN heterostructures. At the same time, several intrinsic polarization‐related properties of NbAlN remain to be determined. In particular, spontaneous polarization, piezoelectric coefficients, and pyroelectric response have not yet been established, and ferroelectric switching behavior has not yet been demonstrated. Therefore, NbAlN should not yet be regarded as a direct replacement for ScAlN in all polarization‐related or ferroelectric functionalities. Rather, the present results establish NbAlN as a metal‐polar, transition‐metal‐containing wurtzite alloy that can be coherently integrated with GaN, retains a wide optical bandgap, and modulates the interfacial carrier density without intentional doping. Determining the intrinsic polarization‐related coefficients and band alignment of NbAlN will be important subjects for future work.

Regarding ferroelectric properties, the NbAlN films in this study did not exhibit polarization–electric field (*P*–*E*) hysteresis loops under the present measurement conditions, in contrast to ScAlN, for which robust ferroelectric switching has been established. The absence of switching behavior in the present samples may reflect differences between the growth conditions and device structures optimized for high‐density 2DEG formation and those required to suppress leakage currents and enable reliable switching measurements. Exploring growth conditions, composition ranges, and device structures suitable for reliable polarization switching measurements remains an important subject for future work.

## 3 Conclusion

This study established NbAlN as a new polar AlN‐based wurtzite alloy that was highly compatible with GaN heterostructures. By demonstrating that Nb could be incorporated into an AlN‐based wurtzite framework while preserving its crystallinity, polarity, and epitaxial coherence, this study extended the accessible chemical space of polar nitrides beyond that of conventional III‐nitride and ScAlN systems.

Notably, NbAlN demonstrated that the polar functionality of wurtzite nitrides was not limited to alloys composed exclusively of conventional group‐III elements. Instead, transition‐metal‐containing polar alloys could be realized when local structural accommodation occurred within a robust wurtzite framework. This finding opens pathways for exploring selected transition metals as alloying elements in polar nitrides, provided that polarity and epitaxial compatibility are maintained during synthesis.

From a device perspective, the demonstrated enhancement of the 2DEG density highlights the potential of NbAlN as a transition‐metal‐containing polar barrier material for GaN‐based electronic heterostructures. This result suggests a pathway for carrier‐density engineering based on polar heterostructure design without intentional doping, although possible contributions from unintentional impurities cannot be completely excluded.

Furthermore, Nb provides an important extension of the AlN‐based polar alloy space from both materials‐design and resource perspectives. While ScAlN has emerged as a representative polar wurtzite alloy, other elements such as Nb and Y may also serve as potential alloying components for extending the material space of polar nitrides. From a resource perspective, Nb also offers practical advantages over Sc because Nb resources are considered sufficient to meet projected demand, whereas Sc is typically produced as a byproduct and rarely occurs in high concentrations [52]. Thus, the present realization of polar wurtzite NbAlN demonstrates that transition‐metal‐containing polar alloys beyond ScAlN can be achieved while maintaining epitaxial compatibility with GaN. This opens new pathways for exploring a broader range of alloy systems in polar nitride materials.

Overall, NbAlN serves as a platform for systematically studying the interplay between the transition‐metal chemistry, local structural accommodation, and polarization in AlN‐based wurtzite alloys. Beyond immediate device applications, these insights can inform the rational design of next‐generation polar nitride materials that integrate electronic, electromechanical, and potentially superconducting quantum functionalities within a single epitaxial material platform for future devices.

## 4 Experimental Section

### 4.1 Epitaxial Growth of NbAlN Thin Films

NbAlN thin films were epitaxially grown on Ga‐polar GaN/sapphire templates using reactive sputter epitaxy. Before deposition, the substrates were thermally cleaned under vacuum. NbAlN was grown in an ultrahigh‐vacuum sputtering chamber evacuated to a base pressure of the order of 10^−8^ Pa using a turbomolecular pump. Independent 2‐inch high‐purity Nb (99.9%) and Al (99.999%) metal targets were employed. While higher‐purity Nb targets could in principle further reduce oxygen‐related impurities, Nb targets with purities substantially higher than 99.9% are not commonly available for sputtering applications. In the present study, high‐purity process gases together with inline gas purification and an ultrahigh‐vacuum growth environment were employed to minimize impurity incorporation, and particular care was taken to suppress oxygen incorporation during epitaxial growth. Reactive sputtering was performed in a mixed atmosphere of high‐purity Ar and N_2_ (99.9999%), both of which were purified using inline gas purifiers. The total growth pressure was maintained in the range of 0.5–1.0 Pa.

Pulsed direct current power supplies were used for both targets. The applied sputtering power was independently adjusted to control the Nb composition while maintaining a constant Al flux (deposition rate). The typical pulse frequencies for the Nb and Al targets were 4 and 10 kHz, respectively. The substrate temperature during growth was varied between 675°C and 800°C, depending on the sample series. The resulting NbAlN film thicknesses were in the range 10–115 nm.

To evaluate the composition dependence of the in‐plane lattice parameters, an additional series of NbAlN films was grown on AlN templates consisting of a thick AlN layer deposited on a sapphire substrate. These templates enabled partial strain relaxation at higher Nb composition and allowed evaluation of the apparent in‐plane lattice constant from RSM measurements.

### 4.2 Composition Analysis

XPS was used to determine the Nb composition of the NbAlN films. The Nb composition was calculated from the integrated intensities of the Nb 3*d* and Al 2*p* core level peaks after background subtraction and correction using standard atomic sensitivity factors.

### 4.3 Structural and Surface Characterization

The surface morphology and epitaxial surface quality after growth were examined using RHEED analysis. The crystal structure and lattice parameters were characterized using high‐resolution XRD, including 2*θ*/*ω* scans, *φ* scans, rocking curves, and symmetric and asymmetric RSM. The epitaxial strain state and coherence with the underlying GaN or AlN templates were evaluated from RSM measurements around the asymmetric reflections.

### 4.4 STEM Characterization

Cross‐sectional STEM specimens were prepared at cryogenic temperature by FIB in‐situ lift‐out using a Helios 660 DualBeam instrument (Thermo Fisher Scientific). The specimens were thinned to electron transparency, and final low‐energy thinning was performed at an accelerating voltage of 2 kV to minimize ion‐beam‐induced damage. Atomic‐resolution STEM observations were performed at Eurofins EAG using an aberration‐corrected Hitachi HD‐2700 microscope operated at an accelerating voltage of 200 kV. ABF‐ and HAADF‐STEM images were acquired to determine the crystallographic polarity and examine the local atomic structure and compositional contrast. Elemental distributions were analyzed by STEM‐EDS.

### 4.5 Atomic‐Column Detection and Local Lattice‐Parameter Analysis From HAADF‐STEM Images

Atomic‐column positions were determined using custom‐written Python code implemented with NumPy, OpenCV, scikit‐image, and SciPy. Initial atomic‐column candidates were identified directly in the unfiltered HAADF‐STEM image by normalized template matching using an 11 × 10‐pixel template cropped around a representative atomic column. Atomic‐column candidates were selected as local maxima in the normalized template‐matching response map, with a minimum separation of 6 pixels and response values of at least 40% of the global maximum response.

A Fourier‐domain background‐suppression filter was subsequently applied to generate an auxiliary lattice‐filtered image containing Fourier components corresponding to the observed wurtzite lattice periodicities. Each candidate position was shifted to the maximum‐intensity pixel within a 7 × 7‐pixel neighborhood in the auxiliary image. This adjustment was used only to obtain stable initial coordinates. The final subpixel positions were determined directly from the unfiltered HAADF‐STEM image by fitting the local intensity distribution within a 17 × 17‐pixel window centered at each snap‐adjusted coordinate to

$$I\;\left(x,y\right)=A\exp\left[-\frac{{\left(x-x_{0}\right)}^{2}}{2\sigma_{x}^{2}}-\frac{{\left(y-y_{0}\right)}^{2}}{2\sigma_{y}^{2}}\right]+B.$$

The Gaussian amplitude *A*, widths σ_*x*_ and σ_*y*_, and background *B* were first optimized with the center position fixed at the snap‐adjusted coordinate. The center position was then refined with the Gaussian shape parameters fixed. Both fitting stages used spatially weighted robust least‐squares optimization with a soft‐L1 loss function. The fitted center was required to remain within a Euclidean distance of 5 pixels from the snap‐adjusted coordinate; otherwise, the snap‐adjusted coordinate was retained. The resulting subpixel coordinates (*x*_0_,*y*_0_) were used for the local lattice‐parameter analysis. The mean atomic‐column intensity was calculated from a 9 × 9‐pixel region of the unfiltered HAADF‐STEM image centered at each fitted subpixel coordinate. Local *c*‐axis and projected *a*‐axis directions were estimated separately around each fitted column from neighboring fitted coordinates. Candidate adjacent columns were selected according to their projected separation along each local direction and their perpendicular deviation from that direction. Two‐sided measurements were used whenever valid neighbors were identified on both sides. A one‐sided value was retained only when the missing side extended outside the image or when a reciprocal nearest‐neighbor relation confirmed the remaining adjacent‐column connection and the opposite adjacent column was unavailable. The local *c*‐axis lattice parameter was obtained from the projected neighbor separations along the local *c*‐axis direction. The local *a*‐axis lattice parameter was calculated from the projected spacing along the local *a*‐axis direction using a factor of $2/\sqrt{3}$.

### 4.6 Electrical Characterization

The electrical transport properties were evaluated using an AlGaN‐based heterostructure template. The substrates consisted of Al_0.2_Ga_0.8_N (approximately 3 nm)/AlN (approximately 1 nm)/undoped GaN heterostructures grown on semi‐insulating 6H‐SiC substrates using MOCVD. NbAlN layers were deposited on the templates as barrier layers for electrical characterization.

Hall effect measurements were performed at room temperature using the van der Pauw geometry. Ohmic contacts were formed by gently pressing indium metal onto the four corners of each sample. To ensure direct electrical contact with the underlying AlGaN layer, the substrate corners were partially masked during the NbAlN deposition, leaving the AlGaN surface exposed in the contact regions. The sheet carrier density and electron mobility were determined from the measured Hall coefficients and resistances, respectively.

## Author Contributions

**Atsushi Kobayashi**: conceptualization, data curation, formal analysis, funding acquisition, investigation, methodology, project administration, resources, supervision, validation, visualization, writing – original draft, and writing – review and editing. **Souta Kurogi**: data curation, formal analysis, investigation, visualization, and writing – review and editing. **Tomoya Okuda**: data curation, formal analysis, investigation, visualization, and writing – review and editing **Riku Kikuchi**: data curation, formal analysis, investigation, visualization, and writing – review and editing. **Yusuke Wakamoto**: data curation, formal analysis, investigation, visualization, and writing – review and editing. **Kouei Kubota**: data curation, formal analysis, investigation, visualization, and writing – review and editing. **Hikaru Sasaki**: data curation, formal analysis, investigation, visualization, and writing – review and editing. **Kazuhisa Ikeda**: data curation, formal analysis, investigation, visualization, and writing – review and editing. **Kanako Shojiki**: data curation, formal analysis, investigation, methodology, resources, software, validation, visualization, and writing – review and editing. **Takahiro Kawamura**: data curation, formal analysis, investigation, methodology, resources, software, validation, visualization, writing – original draft, and writing – review and editing. **Toru Akiyama**: data curation, formal analysis, investigation, methodology, resources, software, validation, visualization, and writing – review and editing. **Takehito Seki**: data curation, formal analysis, investigation, methodology, resources, validation, visualization, and writing – review and editing. **Takuya Maeda**: data curation, formal analysis, investigation, methodology, resources, validation, visualization, and writing – review and editing.

## Conflicts of Interest

The authors declare no conflicts of interest.

## Supporting information




**Supporting File**: adma74756‐sup‐0001‐SuppMat.pdf.

## Data Availability

The data that support the findings of this study are available from the corresponding author upon reasonable request.
