# Supplementary material for: Polar Wurtzite NbAlN: A Transition‐Metal Nitride Alloy for Polarization‐Engineered GaN Heterostructures
Source: Adv Mater. 2026 Sep 3;38(60):e74756. doi: 10.1002/adma.74756 (PMC13712217; doi:10.1002/adma.74756)
Supplement: Supplementary file 1 [file ADMA-38-e74756-s001.pdf]

Supporting Information

Polar Wurtzite NbAlN: A Transition-Metal Nitride Alloy for Polarization-Engineered GaN Heterostructures

Atsushi Kobayashi*, Souta Kurogi, Tomoya Okuda, Riku Kikuchi
Yusuke Wakamoto, Kouei Kubota, Hikaru Sasaki, Kazuhisa Ikeda
Kanako Shojiki, Takahiro Kawamura, Toru Akiyama, Takehito Seki, Takuya Maeda

---

Supplementary Note 1: Additional XRD analysis for possible non-wurtzite inclusions

Additional high-resolution X-ray diffraction (HRXRD) measurements were performed to examine possible non-wurtzite inclusions in the NbAlN films, following the phase-analysis strategy reported for wurtzite YAlN films by Leone et al.[1] Figure S1a shows wide-range symmetric $2\theta/\omega$ scans, measured from 25° to 80°, for NbAlN/GaN films with Nb compositions of 11%, 16%, 22%, 25%, and 37%. The patterns are vertically offset for clarity. The expected reflections from wurtzite NbAlN, GaN, and α-Al_2O_3 were observed. The weak GaN 0003 and α-Al_2O_3 0009 peaks are attributed to multiple-diffraction effects under the present measurement geometry. A weak Al 200 peak was also observed as an instrumental background feature originating from the aluminum sample stage.

No diffraction peaks attributable to a crystalline cubic Nb-rich secondary phase were detected in the wide-range scans. For the 37% Nb film, a weak additional non-$000l$ reflection consistent with wurtzite $(10\bar{1}2)$ was observed. The appearance of this reflection indicates the presence of a minor misoriented wurtzite component at the highest Nb composition. Because the XRD measurement provides an area-averaged structural response, it does not establish a one-to-one correspondence between this component and the individual protruding features observed by AFM.

If a rock-salt Nb-rich phase were to form epitaxially within the wurtzite NbAlN film, its close-packed (111) plane may be oriented parallel to NbAlN(0001) and GaN(0001). The δ-NbN 111 reflection is located close to the wurtzite NbAlN 0002 and GaN 0002 reflections, making the phases difficult to distinguish reliably using a symmetric $2\theta/\omega$ scan alone. Therefore, ϕ-scan measurements were performed for the 22%, 25%, and 37% Nb films using the off-axis δ-NbN $\{002\}$ reflection calculated from the nominal bulk δ-NbN lattice parameter. No ϕ-dependent peaks attributable to epitaxial δ-NbN domains were detected at this specific diffraction condition (Figure S1b).

The lattice parameter of a hypothetical cubic Al–Nb–N phase could vary with composition. Therefore, the absence of a peak at the nominal bulk δ-NbN $\{002\}$ condition excludes only epitaxial cubic domains whose lattice spacing is close to that of bulk δ-NbN and does not by itself exclude every possible compositionally shifted cubic domain. The wide-range 25–80° scans provide a complementary phase survey that does not rely on a single assumed cubic lattice parameter. On the basis of the combined measurements, no crystalline cubic secondary phase was detected within the angular range and sensitivity of the present XRD analysis. Trace cubic inclusions, peaks overlapping the dominant wurtzite or GaN reflections, or domains below the XRD detection limit cannot be completely excluded.

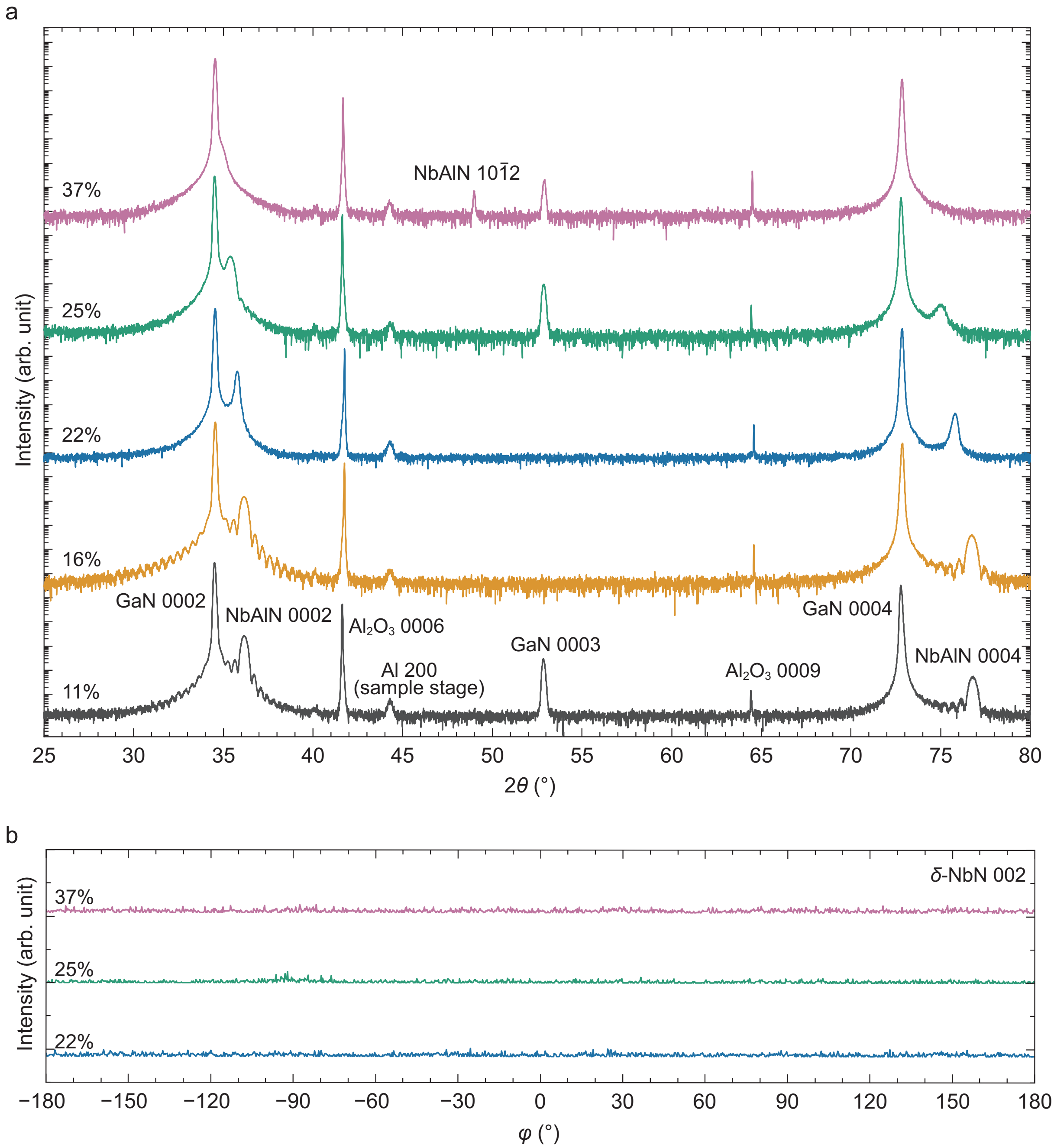

Figure S1: Additional HRXRD analysis for possible non-wurtzite inclusions in NbAlN films. (a) Wide-range symmetric $2\theta/\omega$ scans, measured from 25° to 80°, for NbAlN/GaN films with Nb compositions of 11%, 16%, 22%, 25%, and 37%. The patterns are vertically offset for clarity. A weak additional non-$000l$ diffraction feature consistent with the wurtzite $(10\bar{1}2)$ reflection is observed only for the 37% Nb film, indicating the presence of a minor misoriented wurtzite component. No diffraction peaks attributable to a crystalline cubic Nb-rich secondary phase are detected. The weak GaN 0003 and α-Al_2O_3 0009 peaks are attributed to multiple-diffraction effects. The weak Al 200 peak originates from diffraction by the aluminum sample stage and is not associated with the NbAlN/GaN sample. (b) ϕ-scan measurements for the 22%, 25%, and 37% Nb films at the nominal bulk δ-NbN 002 diffraction condition. No ϕ-dependent peaks attributable to epitaxial δ-NbN domains are detected at this specific diffraction condition.

Supplementary Note 2: Composition Dependence of the *a*-axis Lattice Parameter of NbAlN Evaluated on AlN Templates

In NbAlN films grown pseudomorphically on GaN, the in-plane lattice constant is constrained by the GaN substrate, preventing direct experimental evaluation of the strain-free *a*-axis lattice parameter as a function of Nb composition. To obtain complementary information on the composition-dependent in-plane lattice evolution, an independent series of NbAlN films was epitaxially grown on AlN (approximately 1 μm)/sapphire templates, which provide a smaller in-plane lattice constant than GaN and can promote partial strain relaxation at higher Nb composition.

The film thicknesses of the samples used in this analysis were 61, 40, 64, and 66 nm for Nb compositions of 9%, 10%, 13%, and 17%, respectively. RSM measurements around the asymmetric reflections revealed that NbAlN films with Nb compositions up to 10% were coherently constrained to the AlN template, exhibiting an in-plane lattice constant of approximately 0.311 nm, identical to that of AlN. For Nb compositions exceeding 10%, the NbAlN diffraction peaks shifted along the q_x direction, indicating partial strain relaxation.

The apparent in-plane *a*-axis lattice parameter extracted from these partially relaxed films increased with Nb composition up to 17%. In particular, the 13% and 17% NbAlN films had comparable thicknesses of 64 and 66 nm, respectively, and the 17% NbAlN film exhibited a larger apparent in-plane lattice parameter. This comparison supports the conclusion that the in-plane lattice parameter of wurtzite NbAlN increases with Nb incorporation.

We emphasize that the *a*-axis values extracted from the partially relaxed NbAlN films on AlN templates should not be regarded as fully strain-free bulk lattice parameters, because the degree of relaxation depends on both film thickness and composition. Therefore, Figure S2 is used only to demonstrate the qualitative trend that the apparent in-plane *a*-axis lattice parameter increases with Nb incorporation. The exact strain-free *a*-axis lattice constant and the lattice-matching composition to GaN cannot be determined from these partially relaxed AlN-template data alone.

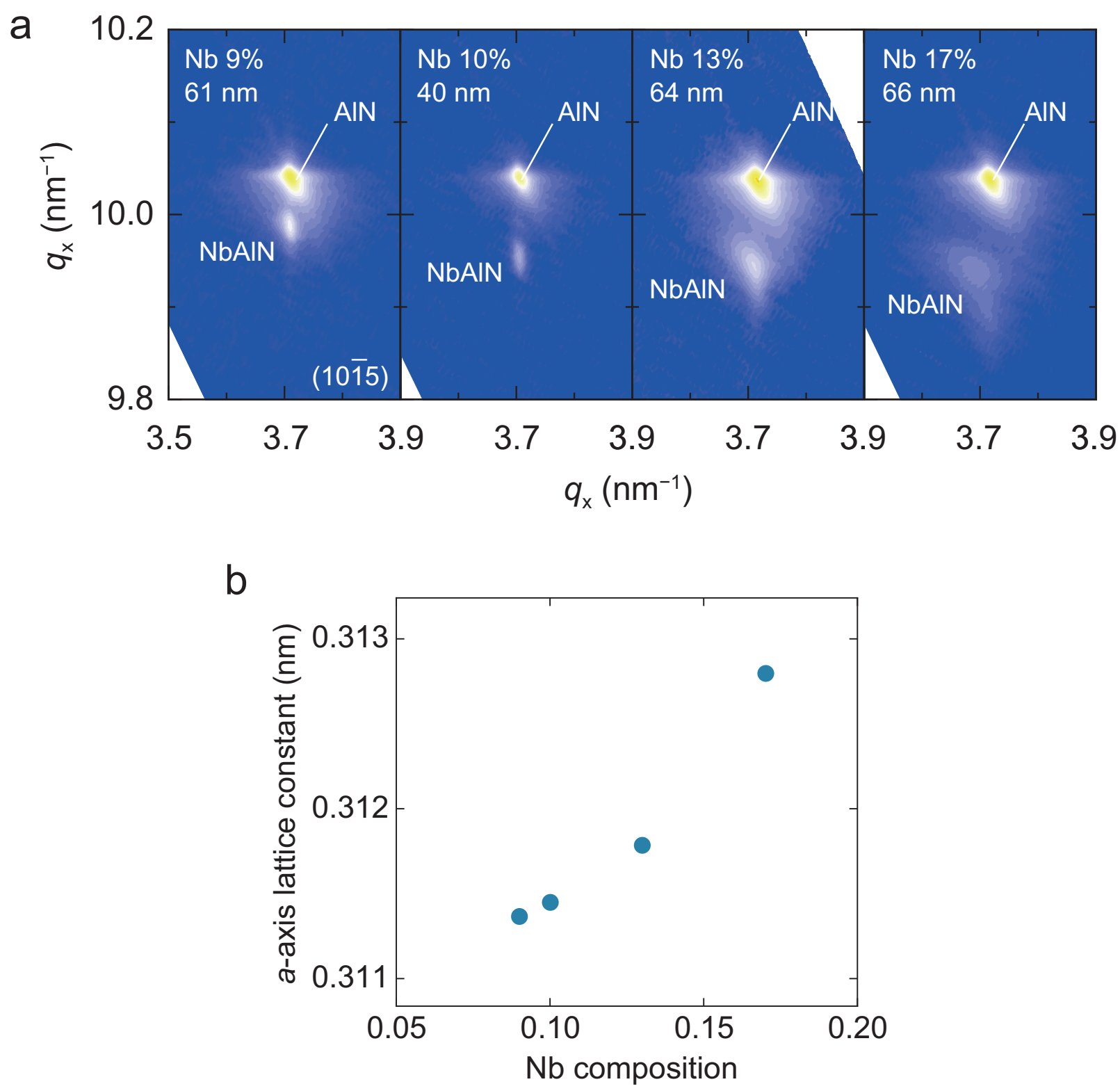

Figure S2: RSM analysis of NbAlN films grown on AlN templates. (a) RSMs around an asymmetric $(10\bar{1}5)$ reflection were measured for NbAlN films with Nb compositions of 9%, 10%, 13%, and 17% and thicknesses of 61, 40, 64, and 66 nm, respectively. For Nb compositions up to 10%, the NbAlN diffraction peaks were aligned with the AlN template peak along the in-plane q_x coordinate, indicating coherent growth with an in-plane lattice constant of approximately 0.311 nm. For Nb compositions exceeding 10%, partial strain relaxation was observed, accompanied by a systematic shift of the NbAlN diffraction peaks along q_x. (b) The apparent in-plane a-axis lattice parameter extracted from the RSMs increased with increasing Nb composition up to 17%. Because the degree of relaxation depends on film thickness and composition, these values should not be interpreted as fully strain-free bulk lattice parameters. Instead, they indicate the qualitative trend of increasing in-plane lattice parameter with Nb incorporation.

Supplementary Note 3: Growth Window and Critical Thickness of NbAlN on GaN

To determine the limits of coherent epitaxial growth, we investigated the structural stability of NbAlN films with varying thicknesses and Nb compositions. Figure S3 summarizes the growth mode map based on the surface observations and RSM. The samples were classified into coherent (crack-free) and cracked/relaxed states.

For lower Nb composition, cracks were observed when the film thickness reached 52 nm, whereas a thinner film with a thickness of 26 nm remained coherent. This early onset of cracking was attributed to the large lattice mismatch between low-Nb-composition NbAlN, which has a smaller in-plane lattice parameter, and the GaN substrates, resulting in significant tensile strain.

As the Nb composition increased, the critical thickness for cracking expanded owing to a reduction in lattice mismatch. Notably, the NbAlN film with 22% Nb maintained a crack-free surface at a thickness of 82 nm, whereas cracks appeared in the 17% Nb film grown to 62 nm, indicating that the stability limit lay within this range for intermediate Nb composition. This trend demonstrates that higher Nb composition not only facilitates strain management but also enables the growth of thick barrier layers required for specific device designs, whereas lower Nb compositions are restricted to thinner layers to prevent tensile failure.

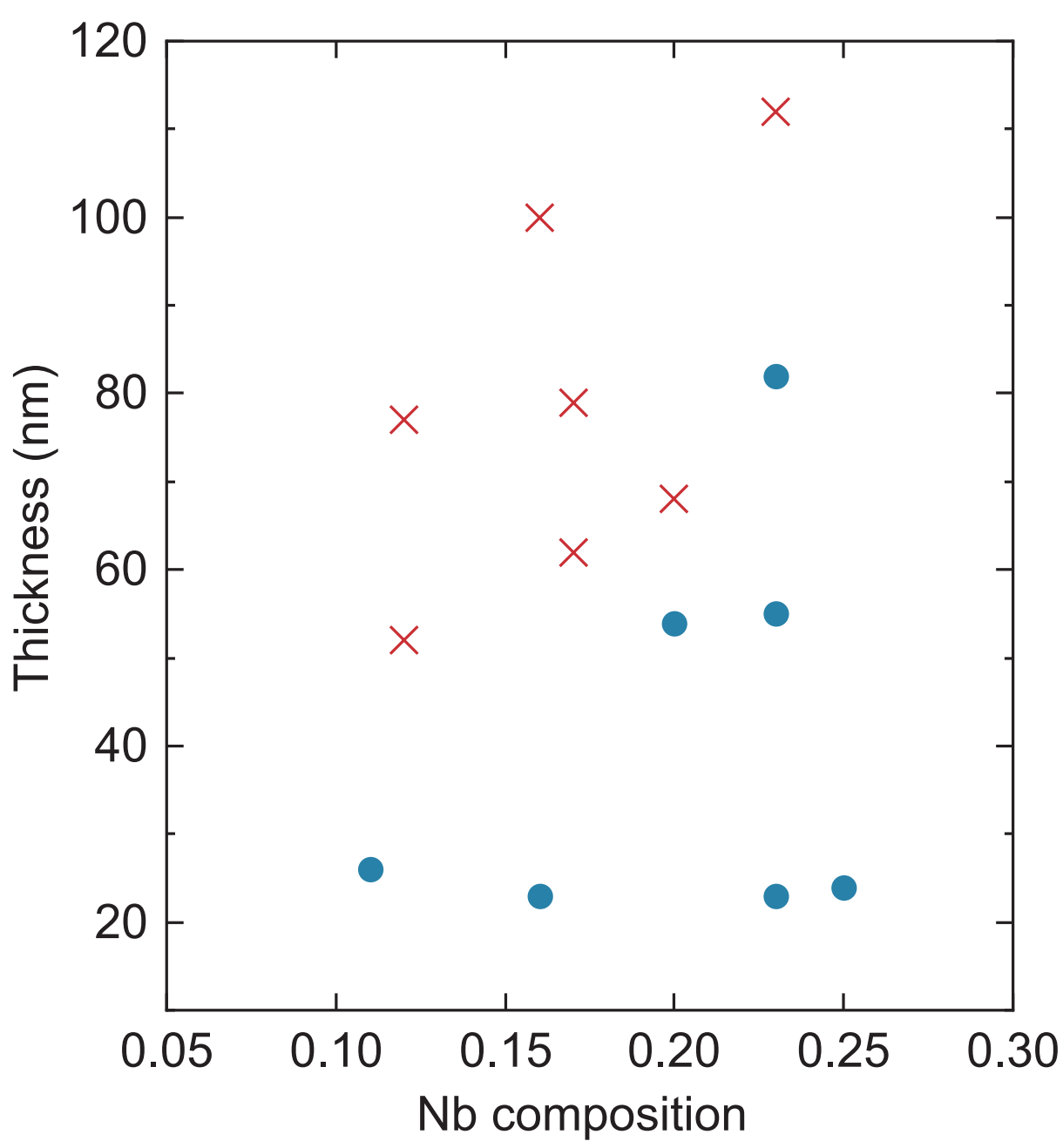

Figure S3: Growth stability map and critical thickness for wurtzite NbAlN films epitaxially grown on GaN substrates. The plot summarizes critical thickness limits for crack formation as a function of Nb composition. Solid blue circles represent coherent, crack-free samples, whereas red crosses indicate samples exhibiting surface cracks or strain relaxation. At lower Nb composition, critical thickness is limited by tensile strain arising from lattice mismatch with GaN, and cracks appear at thicknesses above approximately 50 nm.

Supplementary Note 4: Chemical Bonding State of Nb in NbAlN Investigated by X-ray Photoelectron Spectroscopy (XPS)

XPS measurements were conducted to examine the near-surface bonding environment of Nb and to assess whether a detectable metallic Nb component was present. Figure S4 shows the Nb $3d$ core-level spectrum of a representative wurtzite NbAlN film with a Nb composition of approximately 25%. The spectrum was fitted using two spin–orbit doublets representing a lower-binding-energy Nb–N- or oxynitride-related component and a higher-binding-energy component associated with oxidized Nb species. Because the binding-energy ranges of different Nb oxidation states overlap, these components were not assigned uniquely to specific Nb^{3+} or Nb^{5+} states.

The dominant contribution appeared in the binding energy range of 204.5–204.9 eV, centered at 204.7 eV for Nb $3d_{5/2}$. This peak position is consistent with reported values for Nb-N bonding in niobium nitrides and oxynitrides, indicating that Nb atoms are coordinated with nitrogen within the film. A higher binding energy component observed at approximately 206.5 eV is attributed to surface Nb-O species, which typically originates from the native oxide layer formed during air exposure prior to ex situ measurements.

Notably, no distinct spectral component attributable to metallic Nb (Nb^0) was detected at approximately 202.0 eV within the sensitivity of the near-surface XPS measurement. This result does not, by itself, determine the chemical state of the buried Nb-rich nanoscale regions observed by STEM. These results support the conclusion that Nb substituted into the cation sublattice of the wurtzite structure, forming a coherent NbAlN alloy rather than segregating into metallic inclusions.

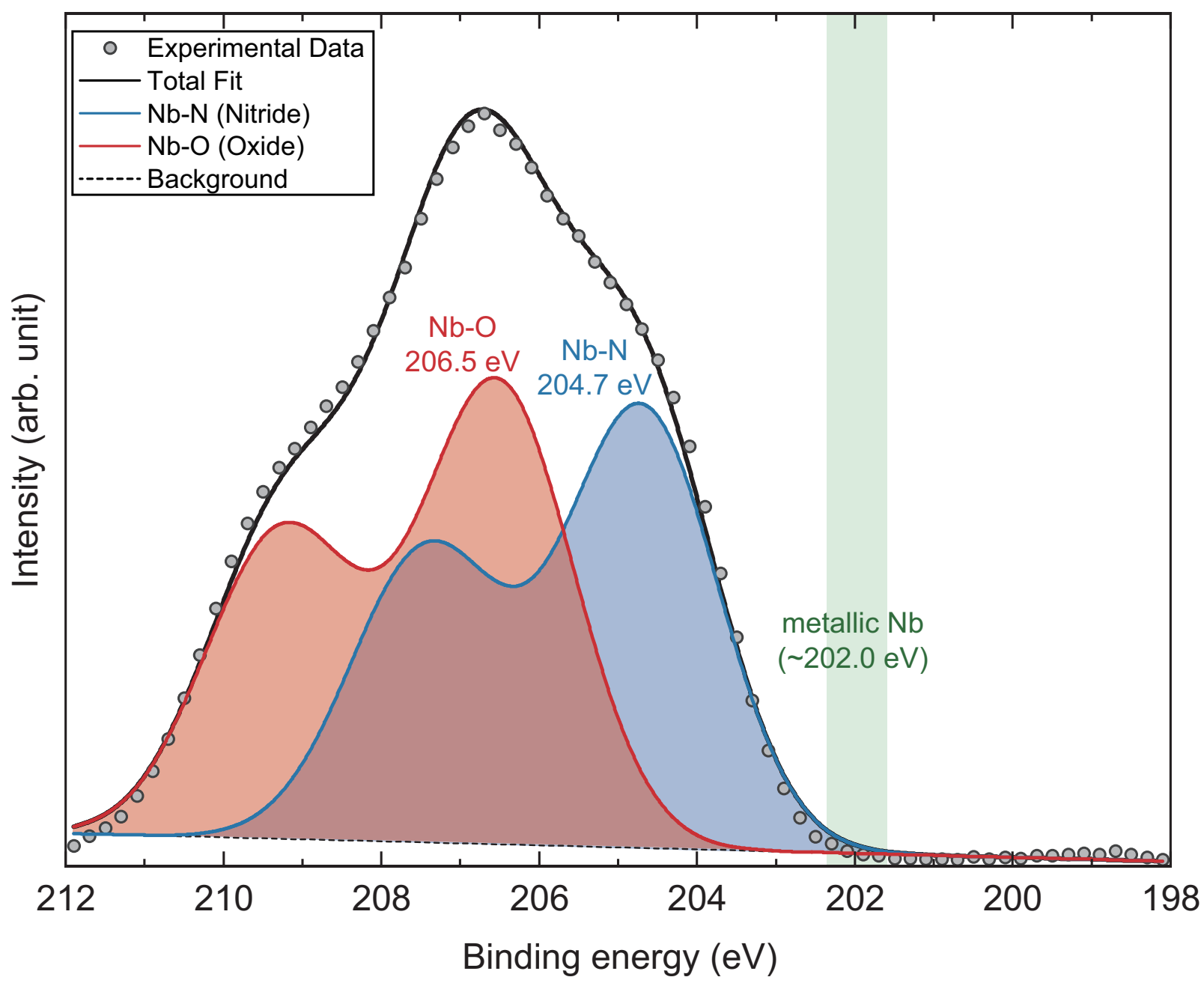

Figure S4: Chemical bonding state of Nb in wurtzite NbAlN films. High-resolution XPS spectrum of Nb $3d$ core level for representative NbAlN film (Nb composition of approximately 25%). Experimental data (gray dots) were deconvoluted into two doublet components using a pseudo-Voigt profile with a linear background. The dominant lower binding energy (blue area) corresponds to Nb-N bonds within the wurtzite lattice, whereas the higher binding energy component (red area) is attributed to surface Nb-O species resulting from air exposure. The green shaded region indicates binding energy range expected for metallic Nb (Nb^0, $\sim$202.0 eV). No distinct spectral component attributable to metallic Nb^0 was detected within the sensitivity of the present near-surface XPS measurement. Because XPS is surface sensitive, these data do not determine the chemical state of the buried Nb-rich nanoscale regions observed by STEM.

Supplementary Note 5: Theoretical Stability of Wurtzite NbAlN Under Epitaxial Strain

To understand the phase stability of NbAlN alloys grown on GaN substrates, we performed first-principles calculations based on density functional theory (DFT) using the Quantum ESPRESSO simulation package.[2] The formation energies $E_f = E_{\mathrm{Nb}_x\mathrm{Al}_{1-x}\mathrm{N}} - xE_{\mathrm{Nb}}^{\mathrm{bulk}} - (1-x)E_{\mathrm{Al}}^{\mathrm{bulk}} - \frac{1}{2}E_{\mathrm{N}_2}$ of the wurtzite and rock-salt phases were calculated for NbAlN alloys with varying Nb compositions, x, where $E_{\mathrm{Nb}_x\mathrm{Al}_{1-x}\mathrm{N}}$ is the total energy per formula unit of the NbAlN alloy, $E_{\mathrm{Nb}}^{\mathrm{bulk}}$ and $E_{\mathrm{Al}}^{\mathrm{bulk}}$ are the total energies per atom of bulk Nb and Al, respectively, and E_{N_2} is the total energy of an N_2 molecule. To simulate the coherent epitaxial growth observed in our experiments, the in-plane lattice parameters of the wurtzite (0001) structure were constrained to match those of the GaN substrate, allowing relaxation only along the out-of-plane (c axis) direction and internal atomic coordinates. Similarly, for the rock-salt structure, the in-plane lattice parameters of the (111) plane were fixed, while relaxation was allowed only along the out-of-plane [111] direction and for the internal atomic coordinates. For comparison, calculations were performed under strain-free bulk conditions and AlN substrate constraints.

The energy difference between the rock-salt and wurtzite structures, $\Delta E = E_f^{\text{rock-salt}} - E_f^{\text{wurtzite}}$, is shown in Figure S5a. In parallel with energetic stability, the structural evolution of the wurtzite lattice was analyzed. Figure S5b shows the calculated c-axis lattice parameters as a function of Nb composition. Under strain-free bulk conditions, the c-axis length exhibits irregular fluctuations and a pronounced decrease at Nb compositions of approximately 40–50%, reflecting structural instability of the wurtzite framework in the absence of epitaxial constraints. This abrupt reduction originates from structural relaxation into a rocksalt-like phase for some initial atomic configurations in the bulk calculations.

In contrast, under coherent epitaxial strain imposed by GaN and AlN substrates, the c-axis lattice parameter increases monotonically with increasing Nb composition. This monotonic expansion indicates that the epitaxial constraint effectively stabilizes the wurtzite structure against spontaneous deformation by accommodating larger Nb atoms through regular expansion along the c-axis. This theoretical trend for growth on GaN substrate is in excellent agreement with the experimental X-ray diffraction results (Figure 2b), corroborating the formation of a coherent wurtzite alloy phase.

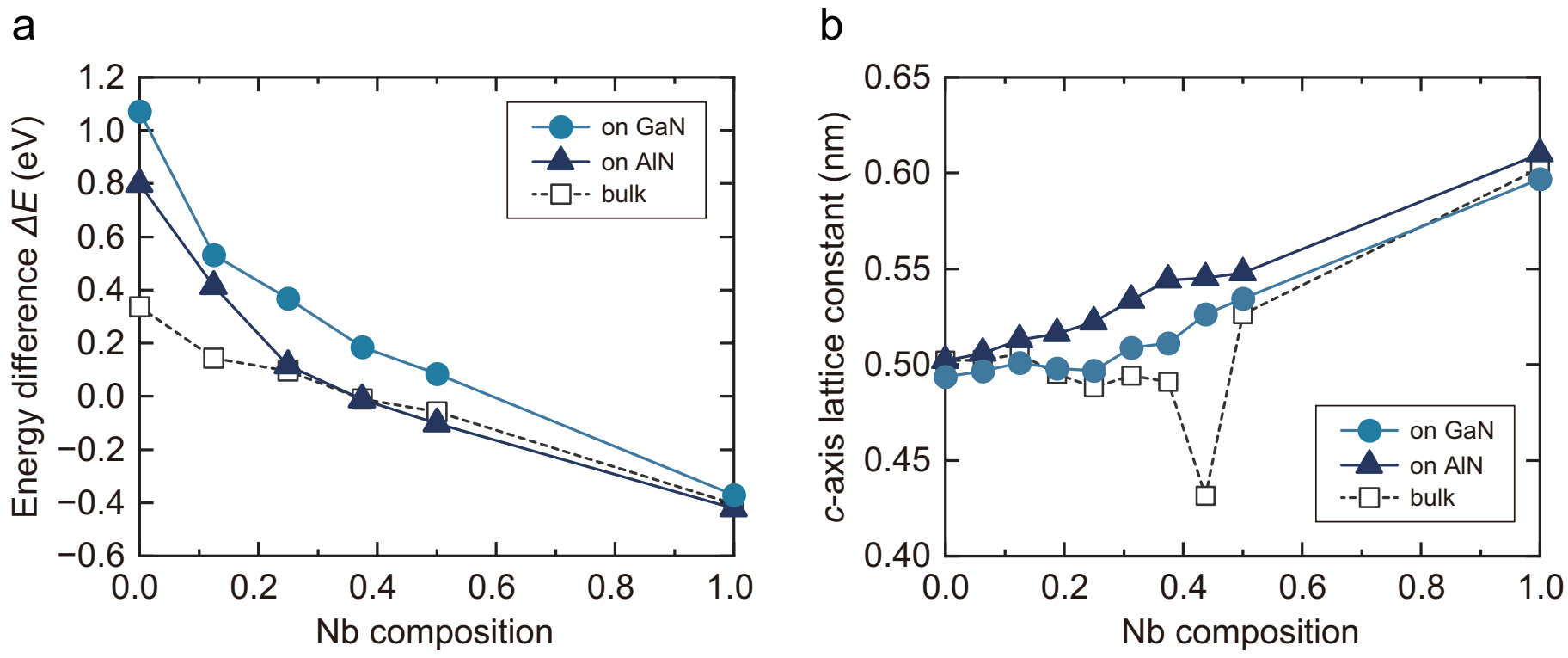

Figure S5: Calculated energy difference and *c*-axis lattice parameters of NbAlN as a function of Nb composition. (a) The calculated energy difference $\Delta E = E_f^{\text{rock-salt}} - E_f^{\text{wurtzite}}$ as a function of Nb composition. A positive ΔE indicates that the wurtzite phase is energetically favored over the rock-salt phase. The results revealed that the moderate strain imposed by the GaN substrate expanded the stability window of the wurtzite phase compared to those under strain-free bulk conditions and on an AlN substrate. Under the coherent epitaxial-strain constraint imposed by GaN, the calculated energy difference between the wurtzite and rock-salt structures changes sign at a Nb composition near 60%, indicating an approximate energetic crossover; the corresponding crossover under strain-free bulk conditions occurs near 35%. This theoretical prediction supports our experimental realization of single-phase wurtzite NbAlN films with Nb composition of up to 25% and suggests that epitaxial stabilization plays a key role in maintaining the wurtzite structure at high Nb composition. (b) The plot compares the values obtained under strain-free bulk conditions with those calculated under coherent epitaxial strain constraints imposed by AlN and GaN substrates. While the bulk calculations show abrupt structural deviations at higher Nb composition, the calculations for coherent growth on GaN and AlN substrates exhibit a monotonic increase in the *c*-axis lattice parameter. Although this behavior depends on the specific atomic configuration considered here, the abrupt deviations observed in the bulk calculations suggest an increasing structural instability at higher Nb compositions.

Supplementary Note 6: Local Bonding Distortions in Wurtzite NbAlN Under Coherent Strain on GaN

To quantify the local structural relaxation induced by Nb incorporation, we analyzed the relaxed atomic configurations of wurtzite $Nb_xAl_{1-x}N$ with $x = 0$, 0.125, 0.25, 0.375, and 0.50. The same coherent epitaxial boundary condition as that used in the phase-stability calculations was applied: the in-plane lattice parameters were constrained to those of GaN, while the c-axis lattice parameter and internal atomic coordinates were relaxed. Cation–N bonds shorter than 2.5 Å were included in the analysis, and the corresponding N–cation–N angles were calculated for each cation site. The standard deviations reported below describe the distributions of local bond lengths and bond angles within each relaxed structure and do not represent uncertainties in the calculated mean values.

Figure S6a shows the average Al–N and Nb–N bond lengths as a function of Nb composition. The average Al–N bond length decreased from 1.934 Å in coherently strained AlN to 1.899 Å at $x = 0.50$. The average Nb–N bond lengths ranged from 2.097 to 2.118 Å and were consistently longer than the Al–N bonds at the corresponding compositions. The bond-length distributions also broadened upon Nb incorporation (Figure S6b). The standard deviation of the Al–N bond lengths increased from 0.007 Å in AlN to 0.024–0.040 Å in the Nb-containing structures, while that of the Nb–N bond lengths ranged from 0.062 to 0.125 Å.

Despite these bond-length variations, the average bond angles remained close to the ideal tetrahedral angle of 109.5° (Figure S6c). The average Al-centered angles were approximately 109.4° across the investigated composition range, whereas the average Nb-centered angles ranged from 106.9° to 108.8°. The bond-angle distributions, however, were substantially broader around Nb atoms (Figure S6d). The standard deviations of the Nb-centered bond angles ranged from 7.0° to 15.1°, compared with 3.9–5.6° for the Al-centered angles in the Nb-containing structures. Thus, the local distortion induced by Nb incorporation is reflected more clearly in the spread of the local geometries than in their mean bond angles. The coexistence of longer Nb–N bonds and broader Nb-centered bond-angle distributions indicates that Nb incorporation is accommodated through heterogeneous local relaxation within the coherently constrained wurtzite framework.

Yazawa et al. reported distinct Al–N and Sc–N bonding environments and substitution-induced local atomic displacements in wurtzite $Sc_xAl_{1-x}N$.[3] The present NbAlN results are qualitatively consistent with this general picture, in which substitution of Al by a transition-metal cation produces chemically differentiated local cation–N bonding environments.

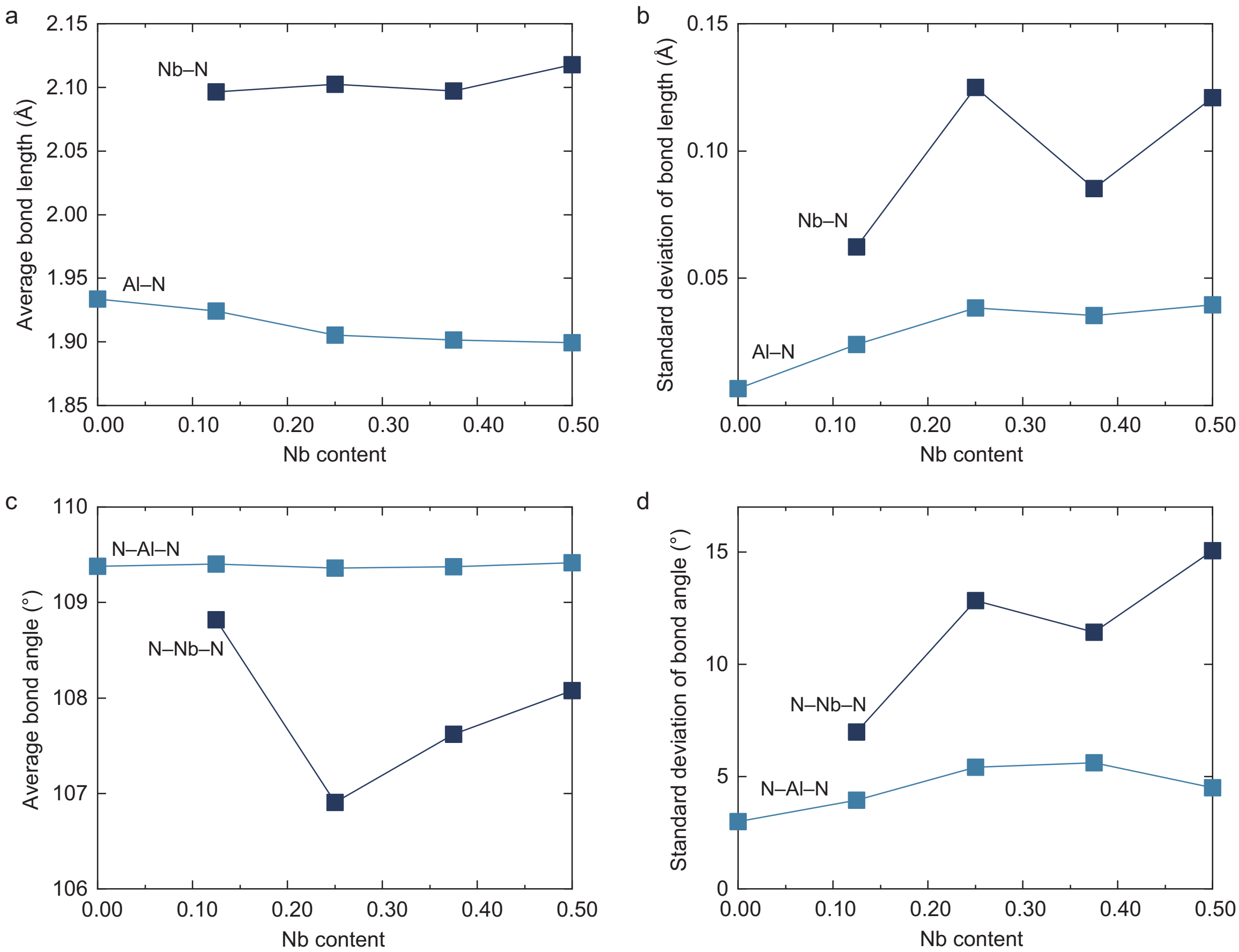

Figure S6: Composition dependence of the local bonding geometry in coherently strained wurtzite $Nb_xAl_{1-x}N$ on GaN. (a) Average Al–N and Nb–N bond lengths. (b) Standard deviations of the Al–N and Nb–N bond-length distributions. (c) Average Al-centered and Nb-centered N–cation–N bond angles. The horizontal dashed line indicates the ideal tetrahedral angle of 109.5°. (d) Standard deviations of the Al-centered and Nb-centered bond-angle distributions. The standard deviations represent the distributions of local geometries within each relaxed structure rather than statistical uncertainties in the mean values. Lines connecting the data points are guides to the eye.

Supplementary Note 7: Polarity of NbAlN near the film surface

To examine whether the polarity of NbAlN is maintained away from the NbAlN/GaN interface, additional atomic-resolution ABF- and HAADF-STEM observations were performed near the upper surface region of the NbAlN layer. Figure S7 shows the corresponding ABF- and HAADF-STEM images. The relative positions of the cation and nitrogen atomic columns in the ABF-STEM image indicate metal-polar stacking, consistent with that observed near the NbAlN/GaN interface in Figure 3b.

No polarity inversion or inversion boundary was observed in the analyzed near-surface region. Together with the interfacial observation, these results indicate that metal polarity is preserved from the interface to the upper region of the NbAlN film within the examined areas.

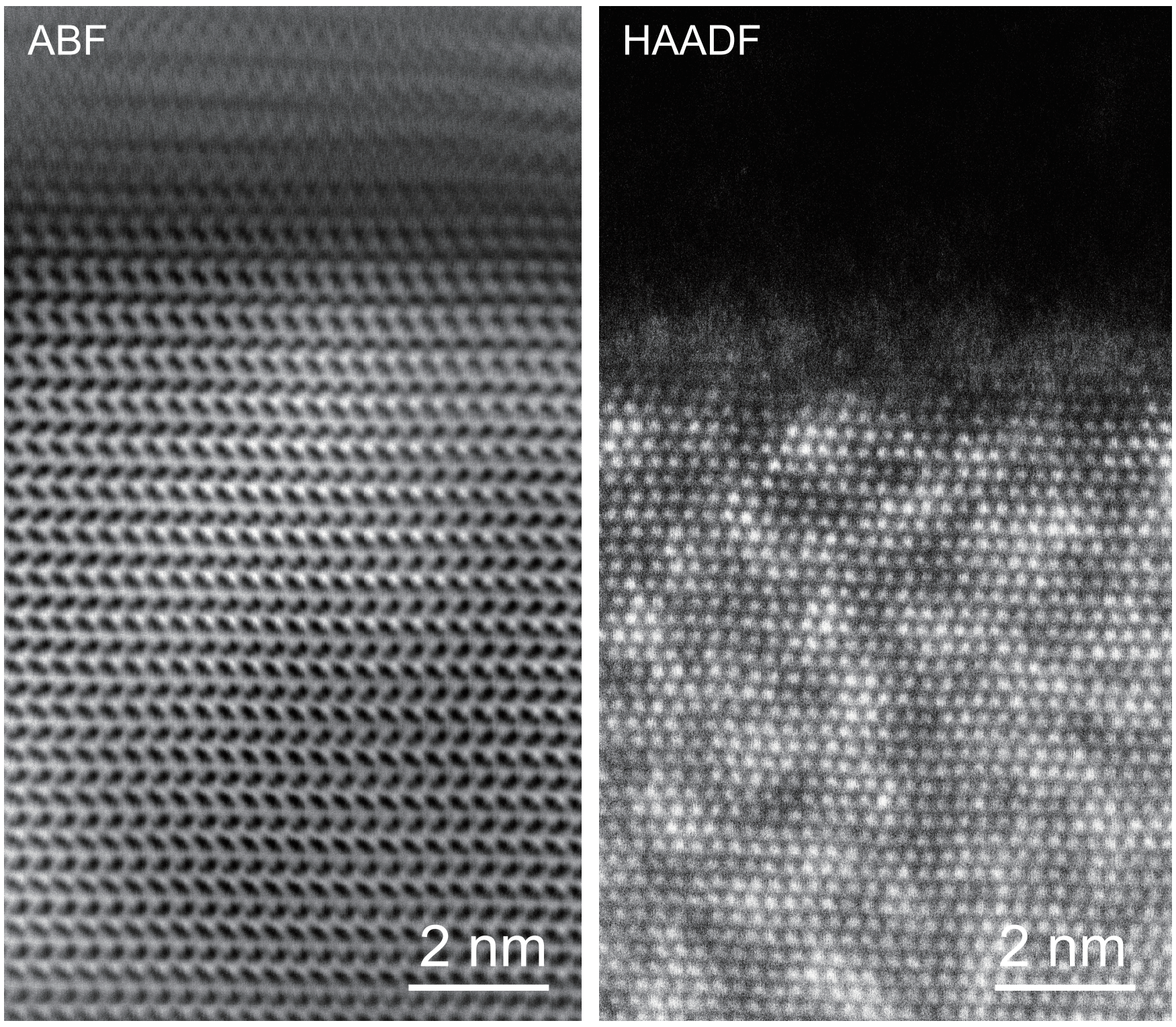

Figure S7: Atomic-resolution STEM observation near the upper surface region of the NbAlN film. ABF- and HAADF-STEM images acquired near the upper surface region of the NbAlN layer. The relative positions of the cation and nitrogen atomic columns in the ABF image indicate metal-polar stacking, consistent with the polarity observed near the NbAlN/GaN interface. No polarity inversion boundary is observed in the analyzed region. Scale bars represent 2 nm.

Supplementary Note 8: STEM-EDS analysis of oxygen distribution at the NbAlN/GaN interface

To assess whether oxygen is present and whether it is localized at the interface between the GaN template and the NbAlN layer, additional STEM-EDS analysis was performed across the NbAlN/GaN interface. Figure S8 shows a HAADF-STEM image of the interfacial region together with elemental maps of Nb, Al, Ga, O, and N. The O elemental map is displayed using an adjusted contrast range to make the weak, spatially distributed counts visible. No thresholding or background-subtraction procedure was applied to suppress the background counts in the revised map.

The Nb, Al, and Ga maps clearly identify the NbAlN layer and the underlying GaN-based template region. A weak O-K signal was observed across the TEM lamella; however, no pronounced oxygen enrichment was apparent either within the NbAlN layer or at the NbAlN/GaN interface. The broadly distributed O signal is consistent with surface oxidation of the TEM lamella rather than localized oxygen accumulation at the buried interface.

Accumulated EDS spectra extracted from the NbAlN region and the NbAlN/GaN interfacial region are also shown in Figure S8. Both spectra contain a weak O-K signal, consistent with the broadly distributed oxygen signal observed in the elemental map. Because STEM-EDS has finite sensitivity for light elements and provides a qualitative assessment over the analyzed region, trace oxygen incorporation at the buried interface cannot be completely excluded. Nevertheless, the present results provide no evidence of pronounced oxygen enrichment at the NbAlN/GaN interface.

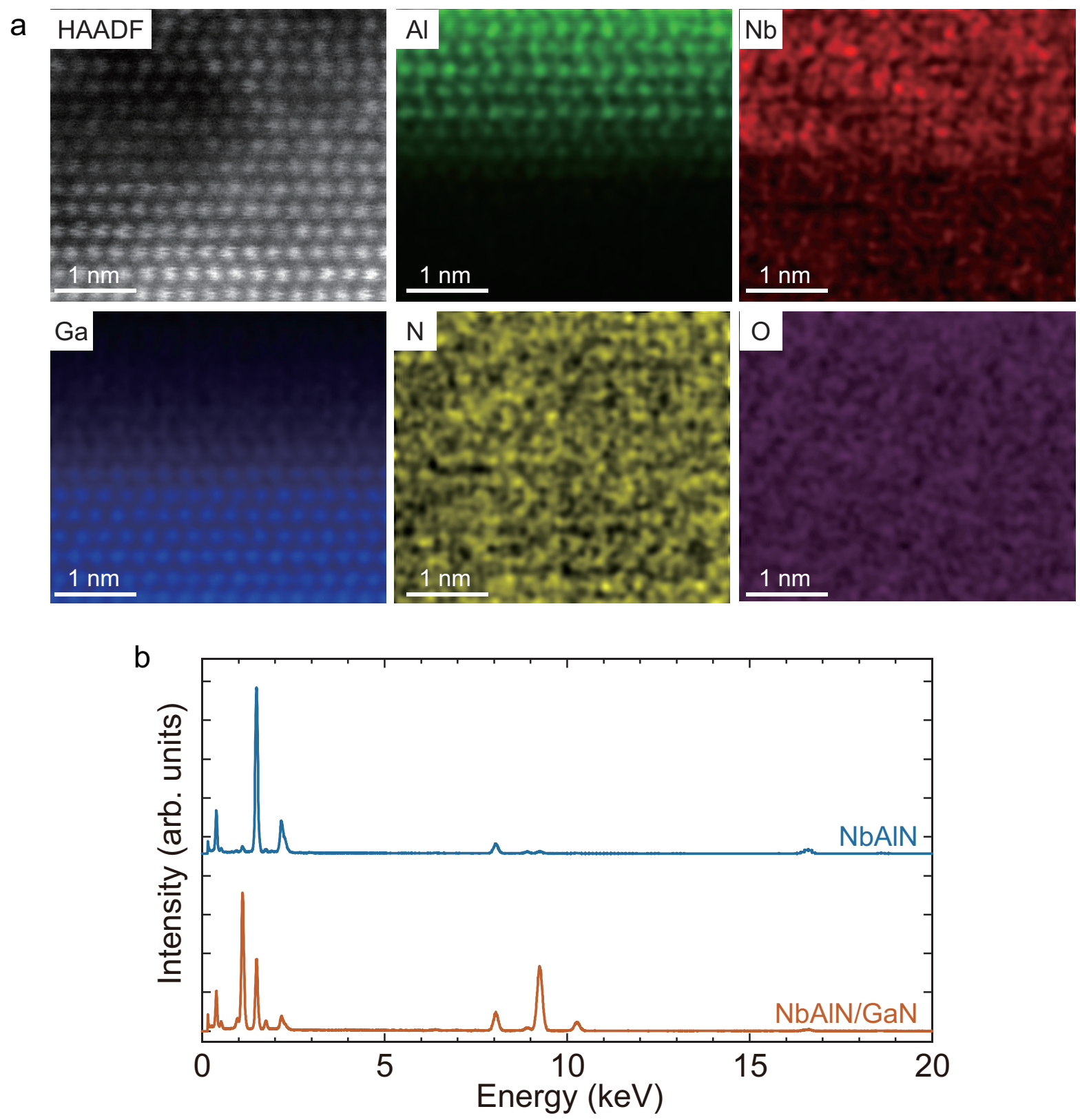

Figure S8: STEM-EDS analysis of oxygen distribution at the NbAlN/GaN interface. (a) HAADF-STEM image of the interfacial region and corresponding elemental maps of Nb, Al, Ga, O, and N. The Nb, Al, Ga, O, and N elemental maps were generated using the Nb Lα, Al Kα, Ga Lα, O Kα, and N Kα signals, respectively. The O-K map shows a weak, broadly distributed signal without pronounced oxygen enrichment within the NbAlN layer or at the NbAlN/GaN interface. (b) Accumulated EDS spectra extracted from the NbAlN region and the NbAlN/GaN interfacial region. Both spectra contain a weak O-K signal.

References

[1] S. Leone, I. Streicher, M. Prescher, P. Stranák, L. Kirste, *Phys. Status Solidi RRL* **2023**, *17*, 2300091.

[2] P. Giannozzi, S. Baroni, N. Bonini, M. Calandra, R. Car, C. Cavazzoni, D. Ceresoli, G. L. Chiarotti, M. Cococcioni, I. Dabo, et al., *J. Phys.: Condens. Matter* **2009**, *21*, 395502.

[3] K. Yazawa, J. S. Mangum, P. Gorai, G. L. Brennecka, A. Zakutayev, *J. Mater. Chem. C* **2022**, *10*, 17557.
